# Three ARID proteins involved in chromatin remodeling PEAT complexes are targeted by the *Ralstonia solanacearum* effector PopP2 and contribute to bacterial wilt disease

**DOI:** 10.1111/tpj.70205

**Published:** 2025-05-13

**Authors:** Léa Monge‐Waleryszak, Maxime Girard, Mélanie Carcagno, Raphaël Culerrier, Céline Vicédo, Yves Martinez, Claire Vérin, Yohann Couté, Valérie Pacquit, Laurent Deslandes

**Affiliations:** ^1^ Laboratoire des Interactions Plantes‐Microbes‐Environnement (LIPME) Université de Toulouse, INRAE, CNRS Castanet‐Tolosan F‐31326 France; ^2^ TRI‐FRAIB Imaging Platform Facilities, FRAIB Université de Toulouse, CNRS, UPS Castanet‐Tolosan 31320 France; ^3^ Univ. Grenoble Alpes, INSERM, CEA, UA13 BGE, CNRS, CEA, FR2048 Grenoble 38000 France

**Keywords:** YopJ acetyltransferase, PopP2, XopJ6, *Ralstonia solanacearum*, *Xanthomonas campestris*, *Arabidopsis thaliana*, ARIDs, PEAT complexes, susceptibility factor, vascular pathogen

## Abstract

Like many gram‐negative phytopathogenic bacteria, *Ralstonia solanacearum* uses a type III secretion system to deliver into host cells a cocktail of effector proteins that can interfere with plant defenses and promote infection. One of these effectors, the nuclear‐targeted PopP2 acetyltransferase, was reported to inhibit many defensive WRKY transcription factors through acetylation. To gain a better understanding of the mechanisms by which PopP2 might exert its virulence functions, we searched for other PopP2‐interacting partners. Here we report the identification of the *Arabidopsis thaliana* AT‐Rich Interaction Domain protein 3 (ARID3) and its close homologs, ARID2 and ARID4, as additional targets of PopP2. These ARID proteins are core components of the chromatin remodeling PEAT complexes that regulate gene expression through histone (de)acetylation and deubiquitination. In yeast, PopP2 binds the conserved C‐terminal part of ARID2/3/4, which contains an α‐crystallin domain putatively involved in their homo‐oligomerization. ARID2/3/4 behave as substrates of PopP2 acetyltransferase activity, which causes the acetylation of several lysine residues conserved between these three proteins and located near their α‐crystallin domain. Interestingly, while PopP2 negatively affects ARID3 and ARID4 self‐interactions *in planta*, it promotes the interaction of ARID3 and ARID4 with PWWP1, another component of PEAT complexes, with which PopP2 can also interact. This study also reveals that disruption of *ARID2/3/4* results in reduced growth of *R. solanacearum*. Overall, our data are consistent with a model in which PopP2 targets several components of PEAT complexes to interfere with their epigenetic regulatory functions and promote *Ralstonia* infection in Arabidopsis.

## Significance Statement

The *Ralstonia solanacearum* effector PopP2 is a YopJ family acetyltransferase. PopP2 is known to dampen basal immune responses by acetylating defensive WRKY transcription factors, and probably by targeting other host components. In this study, we identified three ARID proteins, which are core components of chromatin remodeling PEAT complexes, as additional PopP2 substrates and as factors that promote *Ralstonia* infection in Arabidopsis. Our data suggest that PopP2 may act as a modulator of the assembly of PEAT complexes in order to interfere with their epigenetic regulatory functions.

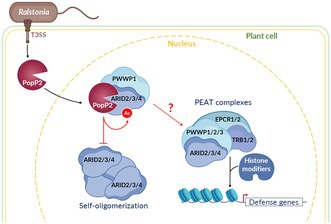

## INTRODUCTION

In nature, microbial pathogens and their hosts are engaged in a co‐evolutionary arms race that is orchestrated by intricate molecular network strategies (Delplace et al., 2022). The plant immune system mostly relies on two layers of pathogen recognition and involves different types of immune receptors. The first layer is activated by cell surface‐localized receptors, known as pattern recognition receptors (PRRs), that can recognize conserved pathogen‐associated molecular patterns (PAMPs) and initiate PAMP‐triggered immunity (PTI). In most cases, this initial layer of defense is sufficient to prevent pathogen colonization (Yuan et al., 2021). However, adapted pathogens deliver virulence effector proteins into host cells, where they operate as immune suppressors by manipulating specific host targets to dampen PTI and promote infection (Wang et al., 2022). To counteract this effector‐triggered susceptibility (ETS), plants have evolved a second layer of the immune system involving intracellular immune receptors, mainly belonging to the nucleotide‐binding domain and leucine‐rich repeat‐containing protein (NLR) family. NLR proteins, through diverse modes of recognition, can sense specific effectors and initiate a robust immune response called effector‐triggered immunity (ETI) that potentiates PTI responses (Ngou et al., 2021; Yuan et al., 2021). In order to get insight into the molecular mechanisms underlying host–pathogen interactions and to develop innovative strategies for combating plant pathogens, it is essential to elucidate the virulence activities deployed by pathogen effectors to promote infection.

Bacterial wilt strains in the *Ralstonia solanacearum* species complex (RSSC) are considered very devastating bacterial pathogens affecting economically important crops worldwide. Indeed, the ability of the RSSC to infect a wide range of host species (over 200), its extensive geographical distribution, and its heightened aggressiveness in warm and humid climates contribute to its status as a significant threat to global agriculture (Vailleau & Genin, 2023). *R. solanacearum* infects its host via the roots and proliferates in xylem vessels where it produces exopolysaccharides that block water and mineral transport, resulting in the development of wilting disease symptoms and ultimately the death of the infected plant (Kai, 2023; Xue et al., 2020). The type III secretion system (T3SS) is one of the main determinants of *R. solanacearum* virulence (Landry et al., 2020). The T3SS is a membrane‐anchored, needle‐like protein complex that enables the bacteria to inject type III effectors (T3Es) directly into host cells (Galán & Waksman, 2018). The strains of the RSSC possess a large repertoire of 50–75 T3Es that interfere with various host functions and immunity to promote infection (Vailleau & Genin, 2023). PopP2, also referred to as RipP2, is one of the best‐characterized *R. solanacearum* T3E as it plays a major role in bacterial virulence and multiplication in several host plants, including tomato, eggplant, beans, and in the model plant *Arabidopsis thaliana* (Le Roux et al., 2015; Macho et al., 2010).

PopP2 belongs to the Yersinia outer protein J (YopJ) T3Es family, hereafter referred to as YopJ T3Es, which are produced by diverse animal and plant bacterial pathogens (Ma & Ma, 2016). The majority of the YopJ T3Es possess a conserved catalytic triad required for their acetyltransferase activity, which is associated with their virulence functions (Cheong et al., 2014; Ma & Ma, 2016). YopJ T3Es use this enzymatic activity to acetylate host proteins on critical residues, thereby affecting their function and/or stability. For example, several YopJ T3Es from animal pathogens, such as YopJ and VopA, inhibit protein kinases associated with the MAPK‐dependent signaling pathway by acetylating key serine and threonine residues in their activation loop, thus leading to the suppression of proinflammatory responses (Ma & Ma, 2016). In plants, the YopJ T3E HopZ3 from *Pseudomonas syringae* acetylates and disrupts the functions of several key immune components (Chakraborty, 2021; Lee et al., 2015). HopZ3 also acetylates other *P. syringae* T3Es to suppress their immune‐inducing activities (Jeleńska et al., 2021; Lee et al., 2015). HopZ1a, another YopJ T3E from *P. syringae*, promotes bacterial infection through acetylation of the jasmonate (JA) ZIM‐domain (JAZ) proteins, lifting the repression of the JA‐dependent signaling pathway and consequently leading to the suppression of salicylic acid (SA)‐mediated defenses (Jiang et al., 2013). In addition, HopZ1a‐triggered acetylation of tubulin causes destruction of plant microtubule networks, disruption of the plant secretory pathway, and suppression of cell wall‐mediated defense (Lee et al., 2012). In *A. thaliana*, PopP2 dampens basal immune responses by acetylating critical lysine residues in the DNA‐binding domain of defensive WRKY transcription factors (TFs), thus inhibiting their *trans*‐regulating functions needed for defense gene expression (Le Roux et al., 2015; Sarris et al., 2015). The same acetylation strategy is used by the *Xanthomonas campestris* pv. *campestris* (*Xcc*) XopJ6 effector, recently identified as the closest homolog of PopP2 within the YopJ family (Lauber et al., 2024). These non‐exhaustive examples highlight the diversity of host components targeted by YopJ T3Es to interfere with eukaryotic processes and promote pathogen infection.

To counteract the virulence functions of YopJ T3Es, some plants have evolved decoy proteins that mimic true effector targets and whose acetylation triggers activation of NLR‐mediated immune responses. For instance, HopZ1a‐triggered immunity involves the Arabidopsis HopZ‐activated Resistance 1 (ZAR1) NLR protein, which can detect HopZ1a‐triggered acetylation of the pseudokinase HopZ‐ETI‐deficient 1 (ZED1), serving as a decoy (Lewis et al., 2010, 2013). In the case of PopP2 and XopJ6, both effectors trigger activation of the Arabidopsis RPS4/RRS1‐R immune receptor complex by acetylating a WRKY decoy domain directly integrated into RRS1‐R (Lauber et al., 2024; Le Roux et al., 2015; Sarris et al., 2015; Williams et al., 2014).

Overall, these findings indicate that YopJ effectors can have multiple targets, but as there is no obvious similarity in the protein sequences of the different substrates identified, it is difficult to predict the number, nature, and function of host proteins that can be manipulated by YopJ proteins. Therefore, to decipher the extent of the virulence functions of YopJ T3Es, a detailed inventory of their substrates is necessary. Hence, to better understand how *R. solanacearum* YopJ T3E PopP2 could exert its virulence functions, we screened by yeast two‐hybrid (Y2H) a cDNA library from *A. thaliana* using PopP2 as bait to identify additional putative PopP2‐interacting partners. This Y2H screening led to the identification of a prey protein corresponding to a truncated form of the AT‐Rich Interaction Domain protein 3 (ARID3). This protein was selected for further characterization as it had not previously been identified as a target of YopJ T3Es.

The ARID protein family constitutes a group of transcription factors and chromatin regulators, characterized by a highly conserved ARID domain, found in a wide range of proteins throughout eukaryotic kingdoms, and that mainly enables the recognition of specific AT‐rich DNA sequences (Korn & Schlundt, 2022). In *A. thaliana*, the ARID family has 10 members divided in to four subgroups depending on the presence of additional domains, besides the ARID domain, in their C‐terminal region (Zhu et al., 2008). ARID3, with its close homologs ARID2 and ARID4, forms the ACD subgroup, so named because they contain an α‐crystallin domain (ACD), also referred to as the Hsp20 domain, which is thought to play a role in protein–protein interactions (Waters & Vierling, 2020). Interestingly, ARID2/3/4 were all shown to be core components of the chromatin remodeling PEAT complexes, which regulate gene expression epigenetically through histone modifications at targeted loci (Tan et al., 2018; Zheng et al., 2023).

These ARID proteins therefore represent promising effector targets that would enable a pathogen like *Ralstonia* to interfere with host epigenetic mechanisms. The targeting of chromatin‐related components by nuclear effectors has been already documented, predominantly in plant–oomycetes and plant–nematodes interactions, as a potent virulence strategy to modulate host transcriptional reprogramming during infection and facilitate host invasion (Chen et al., 2024; Kong et al., 2017; Li et al., 2018; Vijayapalani et al., 2018). Whether and how type III effectors produced by plant bacterial pathogens also target chromatin‐related components remains largely unknown (Harris et al., 2023). Here we investigated the targeting of ARID3 by PopP2 at the molecular level and determined whether its two close homologs, ARID2 and ARID4, are also manipulated by this bacterial effector. Our study reveals that these three ARID proteins behave as direct substrates of PopP2 acetyltransferase activity and that they contribute to *Ralstonia* infection. By influencing protein–protein interactions between components of PEAT, PopP2 is hypothesized to function as a modulator of the assembly of this chromatin remodeling complex.

## RESULTS

### PopP2 interacts with the C‐terminal part of ARID2, ARID3, and ARID4 in yeast

**Figure 1:**
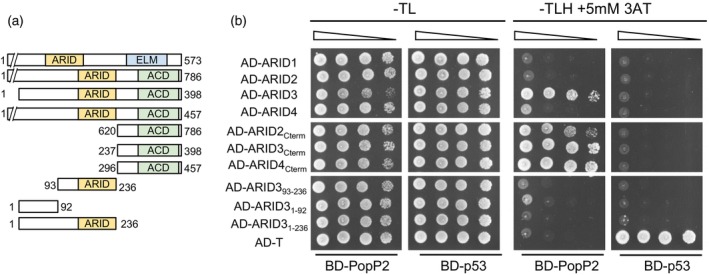
PopP2 interacts with ARID2, ARID3, and ARID4 in yeast. (a) Schematics of full‐length and truncated versions of ARID proteins used in yeast two‐hybrid (Y2H) assays in (b). Proteins were N‐terminally fused with the GAL4 activation domain (AD) and expressed as preys in yeast. The first and last amino acid positions of the different ARID proteins used in the Y2H assay are indicated. The ARID domain is in yellow, the ACD (α‐crystallin domain) is in green, and the ELM domain of ARID1 is in blue. (b) Serial dilutions of yeast cells co‐transformed with different combinations of prey (AD) constructs with PopP2 N‐terminally fused with the GAL4 DNA‐binding domain (BD) as a bait construct. The various AD‐fusion proteins are presented in (a). The murine p53 protein was used as a negative control (BD‐p53) for interaction with AD‐fusion proteins and as a positive control for interaction with the SV40 large T‐antigen (AD‐T). Transformed yeast cells were grown on selective media (SD/−Trp/−Leu [−TL] or SD/−Trp/−Leu/−His supplemented with 5 mm 3‐amino‐1,2,4‐triazole [3‐AT] [−TLH + 5 mm 3AT]). Plates were incubated at 28°C after plating and photographed 5 days later. This experiment was repeated three times with similar results. Production of the different bait and prey proteins in yeast cells was verified by immunoblot analysis (see Figure S1).

PopP2 was previously reported to dampen basal immunity by acetylating a wide range of defensive WRKY TFs (Le Roux et al., 2015). We hypothesized that PopP2 could exert its virulence functions by targeting other nuclear components. To identify such targets, a large‐scale yeast two‐hybrid (Y2H) screening of an *A. thaliana* cDNA library from 10‐day‐old Arabidopsis Col‐0 seedlings was previously performed using PopP2 fused to a GAL4 DNA‐binding domain (BD) as a bait (BD‐PopP2) (Bernoux et al., 2008). This screening identified, among others, a truncated cDNA encoding the last 136 residues of the C‐terminus of the protein ARID3 (residues 237–398 of ARID3, hereafter designated ARID3_Cterm_) (Figure 1). Physical interaction between PopP2 and ARID3 was further confirmed by using full‐length ARID3 fused to the GAL4 activation domain (AD) as a prey protein in Y2H assays (Figure 1). No interaction was detected when ARID3 or ARID3C_term_ were co‐expressed with an unrelated bait protein, the murine p53 protein (BD‐p53). The proper accumulation of the different bait and prey proteins was verified by immunoblot analysis performed on total yeast protein extracts (Figure S1a,b).

Next, we delimited the ARID3 subdomains that could interact with PopP2. For this, three additional AD‐fusion constructs were generated, encoding either the N‐terminus of ARID3 (ARID3_1–92_), the ARID DNA‐binding domain of ARID3 (ARID3_93–236_), or both of them (ARID3_1–236_). Yeast cells co‐expressing BD‐PopP2 with these three additional AD‐constructs did not grow on selective media, suggesting that PopP2 interacts with ARID3 only through binding on ARID3_Cterm_ (Figure 1; Figure S1a,b).

In the ARID family, ARID3 and its close homologs ARID2 and ARID4 form the ACD subgroup (Zhu et al., 2008) and are all components of the chromatin remodeling PEAT complexes (Tan et al., 2018). ARID2/3/4 have variable N‐terminal regions, but their ARID domain and C‐terminal regions, containing an ACD and a loop connecting the ARID and the ACD domains, are conserved. Whether the C‐terminal region of the ARID proteins is a ubiquitous target of PopP2 was therefore evaluated by testing the ability of PopP2 to interact with ARID2 and ARID4 C‐terminus in yeast (Figure 1). A control prey protein corresponding to ARID1, a member of another ARID protein subfamily, devoid of ACD at its C‐terminus, was included to verify the specificity of the interactions detected. Interestingly, whereas no interaction could be detected with full‐length ARID2 and ARID4 in yeast, ARID2_Cterm_ (residues 620–786 of ARID2) and ARID4_Cterm_ (residues 296 to 457 of ARID4) were found to interact with PopP2, as observed with ARID3_Cterm_ (Figure 1; Figure S1a,b). The failure to detect the interaction of full‐length ARID2 and ARID4 with PopP2 in Y2H assays could be due to an incorrect folding of these proteins in yeast cells. These data suggest that ARID2, ARID3, and ARID4 represent host proteins whose C‐terminus part is targeted by PopP2.

Recently, the XopJ6 from *Xcc* that corresponds to the closest homolog of PopP2 in the YopJ family was shown to target and acetylate WRKY TFs, similarly to PopP2 (Lauber et al., 2024). We therefore tested whether XopJ6 could also interact with ARID3. Y2H assays using the catalytic unit of XopJ6 were fused to the BD domain (BD‐XopJ6_224–565_). The results of our Y2H assay indicated that the XopJ6 catalytic unit also binds to ARID3_Cterm_ (Figure S1c,d), thereby suggesting that PopP2 and XopJ6 not only share WRKY TFs as common targets but also ARID3. However, in pursuing this study, we have chosen to focus our investigation on the targeting of ARID proteins by PopP2.

### PopP2 physically interacts with ARID2, ARID3 and ARID4 *in planta*

**Figure 2:**
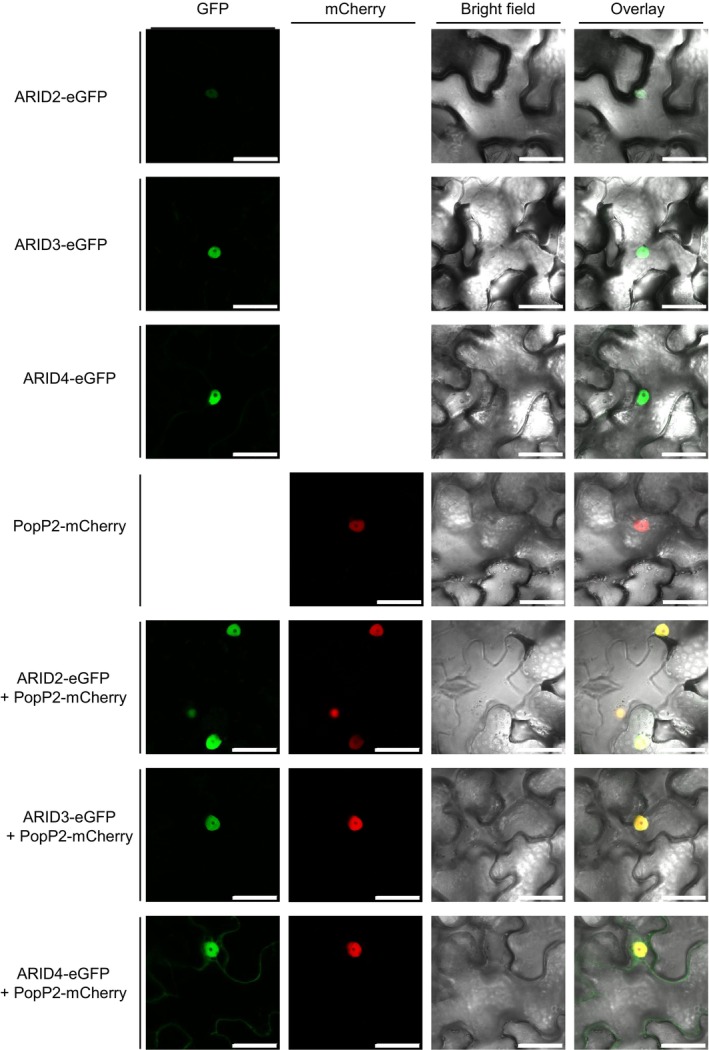
ARID2, ARID3, and ARID4 co‐localize with PopP2 in the plant nucleus. ARID2‐eGFP, ARID3‐eGFP, and ARID4‐eGFP were transiently expressed *in Nicotiana benthamiana* leaves either alone or with PopP2‐mCherry. Confocal fluorescence imaging was performed 48 h post‐agro‐infiltration. Scale bars = 50 μm. The expression of the different fusion proteins in *N. benthamiana* was checked by immunoblot analysis (see Figure S2).

As core components of the nuclear PEAT complexes, the transient expression in *Nicotiana benthamiana* leaves of ARID2, ARID3, and ARID4 C‐terminally fused to enhanced green fluorescent protein (eGFP) confirmed their nuclear localization. Interestingly, unlike ARID2 and ARID3, which accumulate exclusively in the nucleus, ARID4 showed a nucleocytoplasmic localization (Figure 2; Figure S2). The nuclear co‐localization of these ARIDs‐eGFP and PopP2 C‐terminally fused to the mCherry fluorescent protein (PopP2‐mCherry) is consistent with their targeting by the effector in this subcellular compartment.

**Figure 3:**
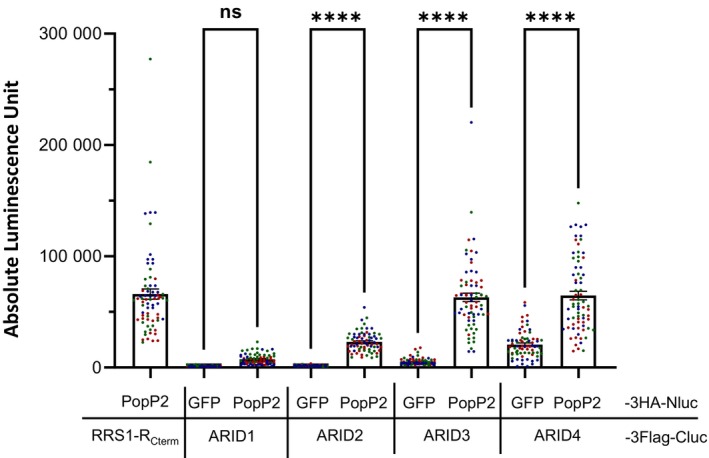
PopP2 physically interacts with ARID2, ARID3, and ARID4 *in planta*. Quantitative measurement of luciferase‐derived signals shows that PopP2 interacts with all three ARIDs in living *Nicotiana benthamiana* cells. For this split luciferase complementation (SLC) assay, PopP2 fused to the N‐terminal part of the luciferase (PopP2‐3HA‐Nluc) was expressed either with GFP or with ARID2/3/4 proteins all fused to the C‐terminal part of the luciferase (X‐3Flag‐Cluc). The co‐expression of PopP2‐3HA‐Nluc with RRS1‐R_Cterm_‐3Flag‐Cluc was used as a positive control for protein–protein interaction. The co‐expression of GFP‐3HA‐Nluc with ARID2/3/4‐3Flag‐Cluc served as negative controls. Leaf samples were collected 48 h post‐agro‐infiltration. Bars indicate mean absolute luminescence unit ± SEM. Three independent replicates were performed (for each replicate, *n* = 24). Data points displayed on the graph are colored depending on the replicate they were collected from. Statistical analysis was performed using one‐way anova with Tukey's test. ****P < 0.0001; ns, non‐significant. The expression of the different fusion proteins in *N. benthamiana* was checked by immunoblot analysis (see Figure S3).

Interaction between ARID proteins and PopP2 was further investigated using a split‐luciferase complementation (SLC) assay enabling the monitoring of protein–protein interactions in living *N. benthamiana* cells. In this assay, two proteins of interest are fused to either the amino‐terminal or carboxyl‐terminal half of luciferase, Nluc and Cluc, respectively. In case of interaction, the two halves of luciferase can be reconstituted to form a functional luciferase enzyme producing luminescence upon addition of its substrate, the luciferin. A triple HA and a triple Flag epitope were added at the N‐terminus of the Nluc and Cluc moieties, respectively, enabling the immunodetection of the transiently expressed proteins. Given that PopP2 binds the C‐terminus of the immune receptor RRS1‐R (RRS1‐R_Cterm_) (Le Roux et al., 2015), co‐expression of PopP2‐3HA‐Nluc with RRS1‐R_Cterm_‐3Flag‐Cluc was used as a positive control (Figure 3). This SLC assay revealed that PopP2 interacts *in planta* with full‐length ARID2, ARID3, and ARID4, but not with ARID1. As negative controls, co‐expression of Cluc‐fused ARID2/3/4 proteins with GFP‐3HA‐Nluc led to significantly lower signals (*P* < 0.0001) than those obtained with PopP2‐3HA‐Nluc. Immunoblot analysis using anti‐Flag antibodies showed that the ARID2 fusion protein accumulates at lower levels than ARID3 and ARID4 fusion proteins (Figure S3), which could explain the lower luminescent signal obtained upon co‐expression of PopP2 with ARID2, compared with those observed with ARID3 and ARID4. Together, these data indicate that PopP2 physically interacts with ARID2, ARID3, and ARID4, likely by targeting their C‐terminus part, as indicated by our Y2H data (Figure 1).

### PopP2 affects the homo‐oligomerization of ARID3 and ARID4 *in planta*

**Figure 4:**
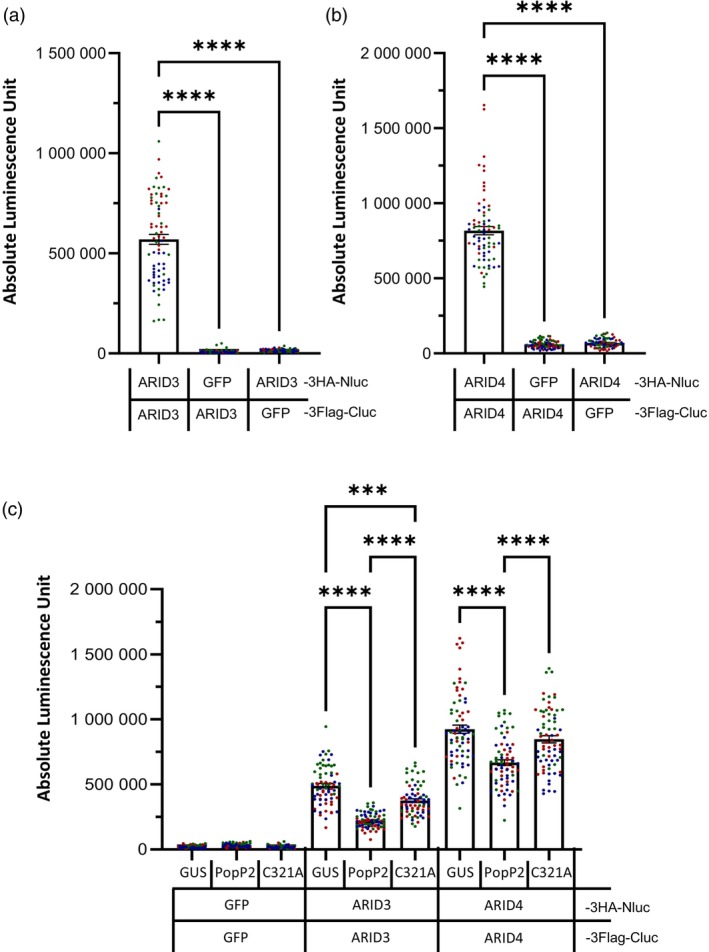
PopP2 negatively affects ARID3 and ARID4 homo‐oligomerization *in planta*. (a, b) Quantitative measurement of luciferase‐derived signals shows that ARID3 and ARID4 homo‐oligomerize *in planta*. Luciferase activity detected in *Nicotiana benthamiana* leaves transiently expressing ARID3 (a) or ARID4 (b) fused to either the N‐terminal part (Nluc) or the C‐terminal part (Cluc) of the luciferase (ARID3/4‐3HA‐Nluc and ARID3/4‐3Flag‐Cluc, respectively). The co‐expression of ARID3/4‐3HA‐Nluc with GFP‐3Flag‐Cluc or GFP‐3HA‐Nluc with ARID3/4‐3Flag‐Cluc served as negative controls. (c) PopP2 affects ARID3 and ARID4 homo‐oligomerization *in planta*. Luciferase activity in *N. benthamiana* leaves transiently expressing ARID3 or ARID4 fused to the N‐terminal part (Nluc) and the C‐terminal part (Cluc) of the luciferase upon transient co‐expression with 3HA‐tagged GUS, PopP2, or C321A. Leaves transiently expressing GFP fused to both Nluc and Cluc in the presence of 3HA‐tagged GUS, PopP2, or C321A served as negative controls. Transient co‐expression with GUS served as a negative control. In (a–c), leaf samples were collected 48 h post‐agro‐infiltration. Bars indicate mean absolute luminescence unit ± SEM. Three independent replicates were performed (for each replicate *n* = 24). Data points displayed on the graph are colored depending on the replicate they were collected from. Statistical analysis was performed using one‐way anova with Tukey's test. ***P < 0.001, ****P < 0.0001. The proper expression of each fusion protein in *N. benthamiana* was checked by immunoblot analysis (see Figure S4).

Previously, the pairwise interactions between several PEAT components revealed that ARID3 and ARID4 self‐interact in yeast (Tan et al., 2018). We therefore investigated whether the targeting of ARID3 and ARID4 by PopP2 could affect their ability to form homo‐oligomers. As a pre‐requisite, we first checked that ARID3 and ARID4 self‐interaction could be monitored *in planta* using the SLC assay. Thus, both full‐length ARID3 and ARID4 proteins were C‐terminally fused with 3Flag‐Cluc or 3HA‐Nluc. As expected, a significant increase in luminescence was detected when ARID3‐3Flag‐Cluc was transiently co‐expressed with ARID3‐3HA‐Nluc (Figure 4a; Figure S4a), indicating that ARID3 forms homo‐oligomers. By contrast, no increase in luminescence was observed upon co‐expression of ARID3 with the negative control GFP in this SLC assay. Similar results were obtained with ARID4 (Figure 4b; Figure S4a).

To investigate whether PopP2 could modulate ARID3 and ARID4 homo‐oligomerization, the Cluc and Nluc fusion proteins of ARID3 or ARID4 were co‐expressed with 3HA‐tagged PopP2 or the unrelated β‐glucuronidase (GUS) protein, serving as a negative control. A significant decrease in luminescence was monitored only when PopP2 was present, suggesting that PopP2 interferes with ARID3/ARID3 and ARID4/ARID4 self‐interactions (Figure 4c; Figure S4b). This could be the result of either direct binding of the effector to their C‐terminal part, thus preventing self‐interactions, and/or a consequence of PopP2 acetyltransferase activity. It is worth noting that PopP2‐C321A can also physically interact with both ARID3_Cterm_ and full‐length ARID3 in yeast, like PopP2 (Figure S4c,d; Figure 1). Interestingly, the reduction of homo‐oligomerization of ARID3 and ARID4 was significantly less severely affected in the presence of a catalytic mutant of PopP2 (PopP2‐C321A). These data suggest that PopP2 acetyltransferase activity plays a role in the PopP2‐triggered reduction of ARID3 and ARID4 self‐interactions.

### PopP2 promotes the association of PWWP1 with ARID3 and ARID4

ARID2, ARID3, and ARID4 were previously shown to directly interact within the PEAT complexes with three PWWP proteins (PWWP1/‐2/‐3) (Tan et al., 2018). These PWWP proteins contain a Pro‐Trp‐Trp‐Pro sequence motif and interact with several histone‐modifying enzymes (Godwin et al., 2024; Tan et al., 2018; Zheng et al., 2023). Since PopP2 affects ARID3 and ARID4 self‐interactions (Figure 4c), we therefore assessed PopP2's impact on ARIDs/PWWP1 interactions. Given that PWWP1 interacts with several PEAT components and particularly with ARIDs with a very high score (Tan et al., 2018; Zheng et al., 2023), we focused our analysis on ARID3/PWWP1 and ARID4/PWWP1 interactions.

**Figure 5:**
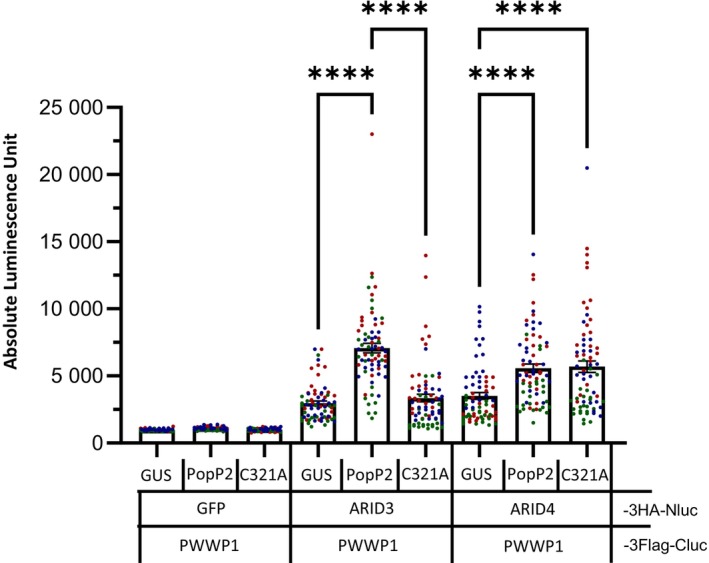
PopP2 promotes the association of ARID3 and ARID4 with PWWP1 *in planta*. Luciferase activity was detected in *Nicotiana benthamiana* leaves transiently co‐expressing ARID3 or ARID4 fused to the N‐terminal part of the luciferase enzyme (ARID3/4‐3HA‐Nluc) together with PWWP1 fused to the C‐terminal part of the luciferase enzyme (PWWP1‐3Flag‐Cluc) upon transient co‐expression with 3HA‐tagged GUS, PopP2, or PopP2‐C321A. Leaves transiently co‐expressing GFP‐3HA‐Nluc and PWWP1‐3Flag‐Cluc served as negative controls. Transient co‐expression with GUS served as a negative control. Leaf samples were collected 48 h post‐agro‐infiltration. Bars indicate mean absolute luminescence unit ± SEM. Three independent replicates were performed (for each replicate *n* = 24). Data points displayed on the graph are colored depending on the replicate they were collected from. Statistical analysis was performed using one‐way anova with Tukey's test. ****P < 0.0001. The proper expression of each fusion protein in *N. benthamiana* was checked by immunoblot analysis (see Figure S5a).

Firstly, the interaction of ARID3 or ARID4 with PWWP1 was verified by using the SLC assay, as shown by an increase of luminescence observed upon co‐expression of PWWP1‐3Flag‐Cluc with either ARID3‐3HA‐Nluc or ARID4‐3HA‐Nluc, but not with the control protein GFP‐3HA‐Nluc. Co‐expression of ARID3‐3HA‐Nluc with PWWP1‐3Flag‐Cluc led to an increase of luciferase activity only in the presence of wild‐type PopP2, but not with PopP2‐C321A or GUS (Figure 5), suggesting that PopP2 enzymatic activity promotes ARID3/PWWP1 association. Interestingly, the SLC assay revealed that while PopP2 also facilitates ARID4/PWWP1 association, this occurs independently of its acetyltransferase activity, as indicated by the increase in luciferase activity resulting from the co‐expression of ARID4‐3HA‐Nluc with PWWP1‐3Flag‐Cluc in the presence of either PopP2 or PopP2‐C321A. In contrast, the two PopP2 variants did not induce any increase in luminescence upon co‐expression of PWWP1‐3Flag‐Cluc proteins with the non‐interacting GFP‐3HA‐Nluc protein, highlighting the specificity of the action of PopP2 on the interaction between PWWP1 and ARID3 or ARID4.

Immunodetection of the different proteins of interest showed that both PWWP1 and ARID3 accumulated at higher levels when co‐expressed with PopP2, but not with GUS or PopP2‐C321A (Figure S5a). Therefore, it is also possible that the increase in luminescence interpreted as a stimulation of PWWP1/ARID3 association by PopP2 is rather the consequence of the enhanced accumulation of PWWP1 and ARID3 induced by wild‐type PopP2. However, the increase in luminescence observed for ARID4/PWWP1 with both PopP2 and PopP2‐C321A was not strictly correlated with an increase of their protein accumulation levels, especially with the catalytic mutant (Figure S5a). Overall, our data strongly suggest that PopP2 does indeed stimulate the interaction of ARID3 and ARID4 with PWWP1, likely in a PopP2 acetyltransferase activity‐dependent manner for ARID3/PWWP1.

Since PopP2 promotes certain ARID/PWWP1 interactions, we speculated that PopP2 might bind and physically stabilize these hetero‐oligomers by a molecular mechanism that remains unknown. Consistent with this hypothesis, a physical interaction between PopP2 and PWWP1 was indeed detected in a Y2H assay (Figure S5b). Taken together, our data demonstrate that PopP2 can bind at least two different types of proteins belonging to the PEAT complexes, likely to interfere with PEAT oligomerization status and functions.

### PopP2 induces acetylation of ARID2, ARID3, and ARID4 on multiple lysine residues *in planta*

**Figure 6:**
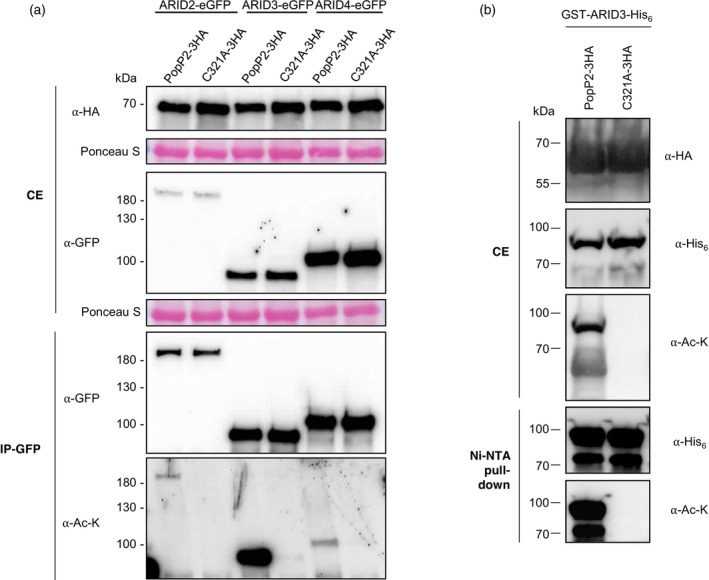
ARID2, ARID3, and ARID4 are acetylated by PopP2 *in planta*. (a) ARID2‐eGFP, ARID3‐eGFP, and ARID4‐eGFP were transiently expressed with 3HA‐tagged active PopP2 or C321A catalytic mutant in *Nicotiana benthamiana* leaves. Samples were collected at 48 h post‐agro‐infiltration. Proteins isolated from crude extracts (CE) were immunoblotted with anti‐HA (⍺‐HA) and anti‐GFP (⍺‐GFP) antibodies. GFP‐tagged ARID proteins were immunoprecipitated on GFP‐trap agarose beads (IP‐GFP) and analyzed by immunoblotting with anti‐GFP (⍺‐GFP) and anti‐acetylated‐lysine (⍺‐Ac‐K) antibodies. Ponceau S staining indicates similar protein amounts loaded in the different lanes. (b) Recombinant GST‐ARID3‐His6 fusion protein was expressed with 3HA‐tagged active PopP2 or C321A catalytic mutant in *Escherichia coli*. Protein extracts (CE) were immunoblotted with anti‐His6 (⍺‐His6) antibodies. GST‐ARID3‐His6 was purified on Ni‐NTA agarose beads (Ni‐NTA pull‐down) and analyzed by immunoblotting with anti‐His6 (⍺‐His6) and anti‐acetylated‐lysine (⍺‐Ac‐K) antibodies. These experiments were conducted three times with similar results.

PopP2 is known to modify the functions of various defensive WRKY TFs by acetylation of critical lysine residues (Le Roux et al., 2015; Sarris et al., 2015). Thus, we tested whether ARID2, ARID3, and ARID4 were acetylated in the presence of PopP2 *in planta*. ARID2, ARID3, and ARID4 fused to the enhanced GFP tag (ARIDs‐eGFP) were individually co‐expressed transiently in *N. benthamiana* with PopP2‐3HA or PopP2‐C321A‐3HA (Figure 6a). As a positive control for this assay, NbWRKY8‐GFP, a known substrate of PopP2 acetyltransferase activity (Le Roux et al., 2015), was used (Figure S6). ARIDs‐eGFP proteins were purified from total plant protein extracts using a GFP affinity matrix. Immunoprecipitated GFP‐tagged proteins were analyzed by immunoblots probed with anti‐GFP and anti‐acetyl‐lysine (anti‐Ac‐K) antibodies. While similar amounts of each GFP fusion protein co‐expressed with PopP2 or PopP2‐C321A were purified, Lys‐acetylated forms of ARID2, ARID3, ARID4, and the control protein NbWRKY8‐GFP were detected only in the presence of catalytically active PopP2, but not with the inactive PopP2‐C321A (Figure 6a; Figure S6). This indicates that PopP2 promotes the acetylation of these three ARIDs on lysine residues.

**Figure 7:**
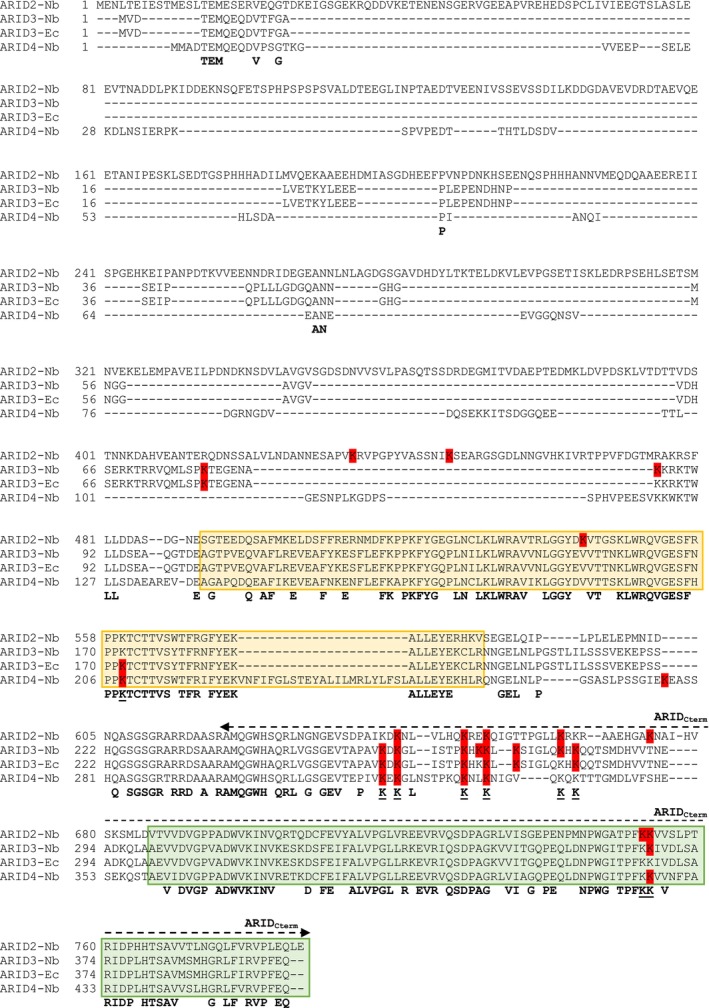
PopP2 causes acetylation of ARID2, ARID3, and ARID4 on multiple lysine residues. Schematic diagram of domain structures and sequence alignment of ARID2, ARID3, and ARID4. Conserved residues in ARID2, ARID3, and ARID4 are indicated in bold. The ARID domain and the ACD are boxed in yellow and green, respectively. ARID_Cterm_ indicates the C‐terminal region of ARIDs shown to interact with PopP2 in the Y2H assay. Acetylated lysine residues reproducibly identified by liquid chromatography–tandem mass spectrometry (LC–MS/MS)‐based proteomics are boxed in red. Conserved lysine residues bearing a PopP2‐induced acetylation are underlined. Mapping of these acetylated sites derived from the analysis of ARID2‐eGFP, ARID3‐eGFP, and ARID4‐eGFP (transiently expressed with 3HA‐tagged active PopP2 or PopP2‐C321A catalytic mutant in *Nicotiana benthamiana* [−Nb]), and GST‐ARID3‐His6 (co‐expressed with 3HA‐tagged PopP2 or C321A in *Escherichia coli* [−Ec]). Purified proteins were digested, and the derived peptides were analyzed by LC–MS/MS. Data were obtained from 2 to 3 replicates for each protein. Acetylated Lys residues identified in peptides with a SC_WT PopP2_/SC_PopP2‐C321A_ ratio ≥0.5 in at least one replicate were considered to be residues preferentially acetylated by active PopP2 (see also Table S1).

To identify the residues acetylated in the presence of PopP2, ARID‐eGFP proteins (ARID2‐, ARID3‐, and ARID4‐eGFP) were produced in *N. benthamiana* leaves with either PopP2‐3HA or PopP2‐C321A and then were immunopurified and subjected to mass spectrometry (MS)‐based proteomic analyses. These experiments revealed several acetylated lysine (Lys) residues in the different ARID proteins, some of them with a higher identification rate in samples co‐expressing PopP2‐3HA compared with PopP2‐C321A‐3HA, with 10, 11, and 7 Lys‐acetylated residues in ARID2, ARID3, and ARID4, respectively (Table S1; Figure 7). Some of them are located just upstream of the ARID domain of ARID2 and ARID3 (ARID2^K434/K447^ and ARID3^K79/K86^), in the ARID domain of ARID2 and ARID4 (ARID2^K542^, ARID4^K208^), and in the ARID2/3/4_Cterm_ (ARID2^K753^, ARID3^K367^ and ARID4^K426^, which corresponds to a lysine conserved in the three ARIDs) (Figure 7). Interestingly, of all the PopP2‐induced acetylation sites identified, nine correspond to Lys residues that are conserved between the three ARID proteins, with eight of them located in the C‐terminal part of ARIDs, consistent with the ability of PopP2 to bind this sub‐region in our Y2H assay (Figure 1). Of these eight residues, six are located in a region between the ARID and the ACD and two are located within the ACD (Figure 7). A limited number of acetylated serine and threonine residues were detected as well, none being specifically identified from samples containing active PopP2 (Table S1), suggesting that PopP2 acetyltransferase activity is not implicated in the modification of these residues, by contrast to the acetylated lysines described above.

### ARID3 is a direct substrate of PopP2 acetyltransferase activity

We next investigated whether PopP2 was able to directly acetylate its interacting ARID partners. For this purpose, we used a bacterial acetylation assay that has previously been successfully used to characterize direct PopP2 substrates. Indeed, expressed in *Escherichia coli*, PopP2 undergoes auto‐acetylation and is capable of acetylating critical Lys residues in the WRKY DNA‐binding domain of WRKY transcription factors in a manner analogous to that observed *in planta* (Le Roux et al., 2015; Tasset et al., 2010). Here, each ARID protein was expressed as a fusion with an N‐terminal glutathione S‐transferase (GST) tag and a C‐terminal six‐histidine residue (His_6_) tag (GST‐ARIDs‐His_6_), together with either PopP2‐3HA or PopP2‐C321A‐3HA. Despite several attempts, only the recombinant GST‐ARID3‐His6 could be produced in a soluble form and with a suitable yield for purification on a Ni‐NTA affinity matrix. Immunoblot analysis of purified GST‐ARID3‐His_6_ revealed the presence of acetylated ARID3 only when co‐expressed with wild‐type PopP2, but not with the catalytically inactive PopP2‐C321A (Figure 6b). The presented data substantiate the fact that ARID3 behaves as a direct substrate of PopP2 acetyltransferase activity.

MS‐based proteomic analyses performed on purified GST‐ARID3‐His_6_ co‐expressed with either active PopP2 or inactive PopP2‐C321A in *E. coli* confirmed PopP2‐dependent acetylation of 7 of the 11 modified lysine residues identified in ARID3‐eGFP co‐expressed with active PopP2 in *N. benthamiana*: K79, K259, K261, K268, K271, K273, and K281 (Figure 7; Table S1). Notably, three of these seven residues (K261, K268 and K271), all located in a predicted loop between the ARID and the ACD domains, were found to be acetylated by PopP2 in the three ARID proteins in *N. benthamiana*. Extrapolation of these data strongly suggests that ARID2 and ARID4, which interact physically with PopP2 (Figures 1 and 3) and which are both acetylated *in planta* in its presence (Figure 6a), could also be considered as direct substrates of PopP2.

The ubiquitous targeting of the conserved lysine residues by PopP2 acetyltransferase activity suggests that the acetylation of the C‐terminal region of the ARID proteins may be critical for PopP2 to manipulate their functions. To explore the effect of Lys‐acetylation on ARIDs functions, we designed an ARID3 acetyl‐mimetic mutant, named ARID3‐11KQ, in which each of the 11 lysines acetylated in the presence of PopP2 was replaced with a glutamine (Q), a polar but uncharged residue classically used to mimic the charge neutralization of acetyl‐lysine. ARID3‐11KQ, like ARID3, physically interacts with PopP2 through its C‐terminal domain in yeast (Figure S7a,b), indicating that the substitutions introduced did not compromise its ability to interact with PopP2. In addition, as expected, acetyl‐mimetic ARID3‐11KQ was no longer detected as being acetylated on Lys residues *in planta* (Figure S7c). However, SLC‐based measurements in living plant cells showed that the interaction between ARID3‐11KQ and PopP2 was disturbed (Figure S7d,e). This discrepancy with the Y2H data could be explained by mislocalization of ARID3‐11KQ. This acetyl mimetic variant indeed exhibited a nucleocytoplasmic distribution, contrasting with the nuclear localization of wild‐type ARID3 and PopP2 as well (Figure S8a,b). This finding suggests that the substitutions introduced impede the capacity of ARID3 to specifically accumulate in the nucleus, thus limiting interactions with nuclear components. In this context, the monitoring of both ARID3‐11KQ self‐association and the reduction of its interaction with PWW1 using SLC assays must be interpreted with caution (Figure S7f–i), as the possible consequences of acetyl‐mimetic mutations on ARID3 interacting properties cannot be dissociated from the mislocalization of the protein that they cause.

### The *arid2/3/4* triple mutant exhibits reduced susceptibility to *R. solanacearum* GMI1000

**Figure 8:**
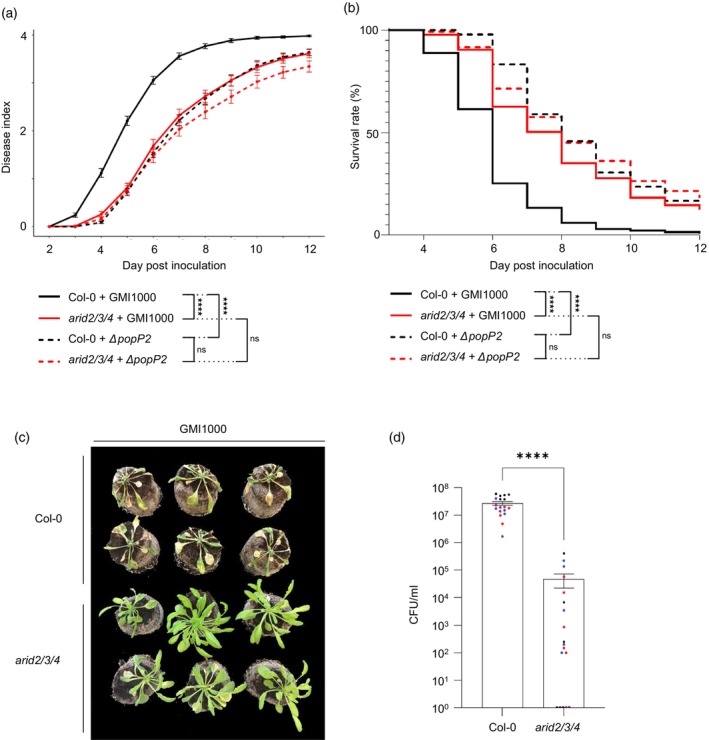
The *arid2/3/4* triple mutant displays reduced susceptibility to *Ralstonia solanacearum* GMI1000. Root inoculation assays were performed on *Arabidopsis thaliana* wild‐type Col‐0 plants and the *arid2/3/4* triple mutant plants using *R. solanacearum* strains GMI1000 or *ΔpopP2*. (a) Disease index (DI) indicating the average wilting symptoms scored daily with a scale from 0 (no symptom) to 4 (100% of wilted leaves). Error bars indicate SEM. Statistical analysis was performed on areas under the curves (AUC) using a pairwise Wilcoxon test. ****P < 0.0001; ns, non‐significant. (b) Survival rate: The disease index (DI) was transformed into binary data to obtain survival rates: DI < 3/4 was defined as “0” (survival) and DI ≥ 3/4 was defined as “1” (death event). Statistical analysis was performed using a Log‐rank test. ****P < 0.0001; ns, non‐significant. DI (a) and survival curves (b) are a composite of four independent biological replicates (*n* ≥ 30 per condition per replicate). (c) Representative phenotypes of *A. thaliana* Col‐0 and *arid2/3/4* triple mutant plants at 8 days post‐inoculation with *R. solanacearum* GMI1000. (d) Growth of *R. solanacearum* GMI1000 in Col‐0 and *arid2/3/4* triple mutant. GMI1000 quantification was determined in the aerial part of root‐inoculated *A. thaliana* Col‐0 and *arid2/3/4*, as described in (a). Bacterial samples were collected for DImean(Col‐0) = 1. The presented plot is a composite from three independent biological replicates (*n* = 6 plants per condition in each replicate). Data points displayed on the graph are colored depending on the replicate they were collected from. Error bars indicate SD. Statistical analysis was performed using a Wilcoxon rank test. ****P < 0.0001.

To determine whether ARID2, ARID3, and ARID4 play a role in plant response to *R. solanacearum*, we used Arabidopsis plants carrying mutations in the genes encoding these proteins. Since ARID2/3/4 have been shown to function redundantly in transcriptional regulation (Tan et al., 2018), we decided to focus our analysis on the phenotypical characterization of the previously published triple mutant *arid2/3/4*. As described previously (Tan et al., 2018), while the development of *arid2/3/4* is stopped at the cotyledon stage in seedlings grown on MS medium, this mutant is able to continue its development up to flowering and seed production when grown in soil. Upon root inoculation of 4‐week‐old plants grown in soil, the *R. solanacearum* GMI1000 strain producing wild‐type PopP2 caused weaker and delayed disease wilting symptoms in the *arid2/3/4* triple mutant compared with wild‐type Col‐0 plants (Figure 8a). Survival curve analysis confirmed this result, showing a significantly higher survival rate for the *arid2/3/4* mutant compared with Col‐0 (Figure 8b,c). Consistent with these observations, plant colonization, measured as bacterial titers in aerial plant tissues, was also severely compromised in the *arid2/3/4* mutant (Figure 8d), indicating that ARID2/3/4 contribute to *R. solanacearum* infection. However, does this contribution depend on the presence of PopP2? We thus investigated the phenotypical response of *arid2/3/4* plants to the Δ*popP2* mutant strain, whose virulence on Col‐0 was reported to be attenuated (Le Roux et al., 2015). Interestingly, the Δ*popP2* mutant did not show additional virulence attenuation in the *arid2/3/4* triple mutant (Figure 8a,b), indicating that abrogation of ARID2/3/4‐dependent functions is deleterious to the development of bacterial wilt disease, regardless of whether PopP2 is produced or not. This leads us to speculate that PopP2 might be manipulating ARID2/3/4 functions to create a physiological context that promotes the pathogenicity of *R. solanacearum*.

## DISCUSSION

The diversity of host plant components targeted by pathogen effectors highlights the sophistication of the virulence strategies employed by phytopathogens to defeat host immune systems. In the repertoire of *R. solanacearum* T3Es, PopP2 is considered a major virulence factor involved both in the virulence and the fitness of this bacterial pathogen (Landry et al., 2020; Macho et al., 2010). There is increasing evidence that PopP2, as several YopJ T3Es, might target multiple host proteins to promote its virulence, in addition to its well‐known targets, the WRKY TFs. Identifying host components targeted by PopP2 is therefore crucial to elucidate its biological activities. In this study, we have identified novel targets of PopP2, namely ARID3 and its two closest homologs in *A. thaliana*, ARID2 and ARID4, which have previously been shown to be part of the PEAT protein complexes that regulate gene transcription through modulation of chromatin condensation (Tan et al., 2018).

### PopP2 acetyltransferase uses ARID2/3/4 proteins as substrates

To demonstrate the physical interaction between PopP2 and ARID2, ARID3, and ARID4, we used two complementary approaches able to detect protein–protein interactions *in vivo*, that is, yeast two‐hybrid (Y2H) and split‐luciferase complementation (SLC) assays. The SLC assays are particularly useful for studying protein–protein interactions in living plant cells because they preserve the native subcellular microenvironment, are highly sensitive, and allow quantitative results to be obtained. Using these two approaches, the physical interaction between PopP2 and ARID2, ARID3, and ARID4 was clearly demonstrated. Furthermore, the C‐terminus of these three ARID proteins has been identified as sufficient for physical interaction with PopP2. This C‐terminal part contains an α‐crystallin domain (ACD) which comprises a compact seven‐stranded β‐sandwich of approximately 90–100 amino acids that forms the structural core of small Heat Shock Proteins (sHSPs) (Waters & Vierling, 2020). Plant sHSPs, originally described as heat shock‐induced, can behave as protein chaperones that oligomerize and bind other proteins, protecting them from various stress‐induced damages (Groenen et al., 1994; Park & Seo, 2015). Due to the massive evolutionary expansion of sHSPs families in plants, ACD‐containing proteins are now numerous and can harbor supplementary domains, hence providing putative additional functions (Scharf et al., 2001; Waters & Vierling, 2020). Whether PopP2 has the ability to target other ACD‐containing proteins or if it does specifically target the ACD of ARID2/3/4 proteins remains to be investigated. Given that PopP2 interacts with and acetylates ARID2/3/4, a MS‐based proteomic analysis was conducted on immunoprecipitated ARIDs co‐expressed *in planta* with catalytically active or inactive PopP2. Although members of the YopJ family can target and modify a range of host components by acetylating specific serine, threonine, and/or lysine residues (Ma & Ma, 2016), ARID2/3/4 were found to be preferentially acetylated in the presence of the active PopP2 only on lysine residues. Interestingly, many of these acetylated lysine residues are conserved in ARID2/3/4 and map to a region located between the ARID and ACD domains. It is noteworthy that three of these conserved lysines were also identified as targeted by active PopP2 in recombinant ARID3 produced in *E. coli*, thereby substantiating their direct acetylation by PopP2. Furthermore, one additional conserved lysine residue, located within the ACD of ARID2/3/4, was found to be acetylated by PopP2 *in planta*. Collectively, these data indicate that ARID2/3/4 represent previously undescribed direct substrates of PopP2 enzymatic activity.

Acetylations are post‐translational modifications that can significantly alter the function of a protein by modifying its properties (i.e. hydrophobicity, solubility, and surface characteristics). These modifications may influence protein conformation, stability, subcellular localization, and interactions with other molecules (Christensen et al., 2019; Finkemeier et al., 2011; Xing & Poirier, 2012). PopP2 was reported to dampen basal immune responses through acetylation of key lysine residues in the WKRY DNA‐binding domain of multiple defensive WKRY transcription factors, causing inhibition of their *trans*‐regulating function involved in defense gene expression (Le Roux et al., 2015). Hence, PopP2 acetylation of conserved lysine residues in the C‐terminal region of ARID2/3/4 suggests that PopP2 also employs this mechanism to interfere with ARID2/3/4 properties. It is therefore tempting to speculate that acetylation of critical lysine residues near and within the ACD of ARIDs could trigger a conformational change that alters their DNA‐binding properties, which would be reminiscent of what has been described for the WRKY TFs acetylated by PopP2. PopP2 could also use acetylation to affect their ability to interact with other nuclear proteins, and in particular those that are part of the PEAT complexes.

### PopP2 interacts with several components of PEAT complexes

Given that ARID2/3/4 proteins have previously been shown to self‐interact, probably through their ACD (Bondino et al., 2012; Li et al., 2015; Waters & Vierling, 2020), and also to associate with PWWP proteins of PEAT complexes (Tan et al., 2018), we investigated the effect of PopP2 on these protein–protein interactions. Due to the difficulty of expressing ARID2 at a sufficient level in *N. benthamiana*, we focused our analysis on ARID3 and ARID4. Interestingly, the SLC assay revealed that ARID3 and ARID4 homo‐oligomerization was more affected in the presence of active PopP2 than with the catalytically inactive PopP2 mutant. As both PopP2 variants are able to physically interact with ARID3, and considering that ARID3 was identified here as a direct substrate of PopP2, our results support a model in which PopP2 acetyltransferase activity interferes with ARID3 and ARID4 homo‐oligomerization. In addition, our SLC assays confirmed the previously reported interactions between PWWP1 and ARID3/4 (Tan et al., 2018; Zheng et al., 2023), and showed that PopP2 promotes the interaction of PWWP1 with both ARID3 and ARID4. Furthermore, PWWP1, which interacts with PopP2 in the Y2H assay, might represent an additional effector target within PEAT complexes. These PopP2‐facilitated interactions between PWWP1 and ARIDs differ slightly depending on the ARID protein and appear to depend not only on PopP2 acetyltransferase activity but also on the presence of the effector, whether catalytically active or not. We hypothesize that PopP2, apart from its enzymatic activity that would modulate these interactions through acetylation of particular residues, could also promote ARIDs/PWWP1 associations simply by physically interacting with these proteins. To evaluate the role of acetylated lysine residues on the interaction properties of ARID3, we generated an acetyl‐mimetic ARID3‐11KQ mutant in which the 11 lysine residues acetylated by PopP2 *in planta* were replaced by glutamines (K → Q). This mutant was included in SLC assays to investigate its ability to interact with both PopP2 and PWWP1, and to self‐associate. Unfortunately, the nucleocytoplasmic localization of ARID3‐11KQ makes it difficult to determine the precise effect of the introduced mutations on ARID3 interaction properties. For example, the lower level of interaction measured between ARID3‐11KQ and its nuclear partners could simply be due to a reduction in the nuclear pool of ARID3‐11KQ compared with that of ARID3. Since the nuclear localization of wild‐type ARID3 seems not to be affected in the presence of active PopP2, it is very likely that the mislocalization of ARID3‐11KQ is caused by the substitution of lysine residues that are critical for its nuclear accumulation, rather than by its acetyl mimetic state. However, we cannot exclude the possibility that multiple forms of ARID3 acetylated by PopP2 on a range of lysine residues coexist in the nucleus, where only a small fraction of ARID3 acetylated on 11 lysine residues at a time would be impaired in its ability to accumulate in the nucleus. Whether the mislocalization of ARID3‐11KQ has any biological significance would require further investigation.

Based on limited structural data currently available, it was not possible to confidently predict the structure of the ARID3 variants or that of a multi‐protein complex involving ARID3, PWWP1, and PopP2. Overall, further structure–function studies are needed to precisely identify the Lysine residues critical for ARID2/3/4 functions and the biological consequences of these acetylations.

It is noteworthy that the well‐characterized BRIGHT protein, that belongs to a sub‐group of ARID proteins in animals, can also self‐associate through its C‐terminal part located downstream of its ARID domain (Wilsker et al., 2005). Interestingly, BRIGHT was shown to form homo‐dimers, while it does exist as a tetramer upon DNA binding (Herrscher et al., 1995; Nixon et al., 2004). Similarly, the C‐terminal part of the ARID protein LjSIP1 from *Lotus japonicus*, a close homolog of ARID2/3/4, is required for LjSIP1 self‐association and association with other proteins (Zhu et al., 2008). LjSIP1 was hypothesized to form a dimer in the absence of its target loci (Zhu et al., 2008). It is therefore tempting to hypothesize that PopP2 could manipulate PEAT functions through regulating ARID2/3/4 oligomerization levels and additionally modulate the subsequent associations with other PEAT components like PWWP1.

### Do PEAT complexes serve as hubs of pathogen effectors to promote host infection?

To investigate the contribution of ARIDs in *A. thaliana* infected by *R. solanacearum*, the *arid2/3/4* mutant previously described by Tan et al. (2018), was root‐inoculated with the GMI1000 strain. The reduced susceptibility of the *arid2/3/4* mutant to the bacteria indicates that ARID2/3/4 contribute to *Ralstonia* infection and should therefore be considered as susceptibility factors to this bacterial pathogen. Given that PopP2 affects two components of the PEAT complexes, it is worth considering that PEAT complexes may be involved in the virulence of *R. solanacearum*. In *A. thaliana*, the PEAT complexes transcriptionally regulate over 8000 genes by recruiting the histone acetyltransferases HAM1/2 and histone deacetylases HDA6/9 to regulate histone H4 acetylation levels and, subsequently, chromatin accessibility at these specific loci (Tan et al., 2018; Zheng et al., 2023). Transcriptomic data generated from 12‐day‐old seedlings of null mutants in PEAT components showed that among the 8000 genes differentially regulated by the PEAT complexes at early stages of plant growth, around a few hundred genes are involved in defense responses (Tan et al., 2018; Zheng et al., 2023). Although our root‐inoculation assays were performed at a later stage of development (e.g. 4‐week‐old plants), we hypothesize that the hijacking of these chromatin‐remodeling complexes by an effector like PopP2 would represent a potent virulence strategy, allowing *R. solanacearum* to modulate the expression of host genes to favor infection. The exact number and nature of the genes modulated by PEAT complexes in infected plants remains unknown and requires further investigation.

A substantial body of evidence derived from a diverse array of pathogens indicates that manipulation of host epigenetic regulation represents a conserved strategy to dampen host immune responses (Harris et al., 2023; Schator et al., 2021). In plants, a few effectors able to modify chromatin condensation were recently discovered in nematodes, oomycetes, and fungi (Harris et al., 2023). For example, the nematode *Heterodera schachtii* effector 32E03 epigenetically regulates plant gene expression by direct inhibition of the *A. thaliana* histone deacetylase AtHDT1 involved in rRNA transcriptional regulation through chromatin modifications (Vijayapalani et al., 2018). The oomycete *Phytophthora sojae* PsAvh52 effector interacts with the soybean histone acetyltransferase GmTAP1 and relocates it to the nucleus, where it can promote transcriptional activation of putative susceptibility genes through the acetylation of histones H2A and H3 (Li et al., 2018). By contrast, the *P. sojae* effector PsAvh23 targets a component of a histone acetyltransferase (HAT) complex to suppress histone H3 acetylation and the activation of defense genes, promoting pathogen infection (Kong et al., 2017).

Pathogen interference with the PEAT complexes might represent a virulence strategy used by at least two different vascular phytopathogenic bacteria. Indeed, the Xop6 effector from *Xcc* was identified here as interacting with ARID3, like PopP2. This suggests that these two closely related YopJ T3Es share common host targets in addition to the defensive WRKY TFs (Lauber et al., 2024). However, it remains unclear whether XopJ6 manipulates ARID proteins in a similar manner and whether it is also able to target other components of the PEAT complexes. More generally, whether PEAT remodeling complexes serve as hubs for phytopathogen effectors to promote infection is a key question to be addressed in the near future.

## EXPERIMENTAL PROCEDURES

### Bacterial strains, yeast strains, and growth conditions

*Agrobacterium tumefaciens* strains were grown at 28°C in liquid YEB medium supplemented with adequate antibiotics: gentamicin (15 μg ml^−1^) and carbenicillin (25 g ml^−1^) for pAM‐PAT‐35S transformed GV3103 strains; gentamicin (15 μg ml^−1^) and spectinomycin (50 μg ml^−1^) for pB7‐35S GV3101 transformed strains; tetracyclin (5 μg ml^−1^) and Kanamycin (25 μg ml^−1^) for pBIN transformed C58C1 strains. *E. coli* Rosetta (DE3) strains (Sigma Aldrich, Darmstadt, Germany) were grown at 37°C in Luria‐Bertani rich medium supplemented with adequate antibiotics. *R. solanacearum* strains were grown for 3 days at 28°C on solid φ medium supplemented with 5 g L^−1^ glucose and 0.004% triphenyl tetrazolium chloride for GMI1000, and also supplemented with gentamicin 7.5 μg ml^−1^ for Δ*popP2*. Strains were then grown in liquid φ medium at 28°C under shaking to OD_600nm_ = 1 before dilution for inoculation assays at OD_600nm_ = 0.01 (10^7^ CFU ml^−1^). For yeast‐two‐hybrid assays, the *S. cerevisiae* AH109 strain was used (Takara Bio, San Jose, CA, USA).

### Plant material

*Nicotiana benthamiana* plants were grown on soil at 21°C and 60% relative humidity, under long day conditions (16 h light/8 h dark). *Agrobacterium tumefaciens*‐mediated transient expression experiments were performed on leaves of 4‐week‐old plants. *Arabidopsis thaliana* plants Col‐0 and *arid2/3/4* triple mutant were grown on Jiffy‐7 substrate pellets (Jiffy Products International AS, Norway) at 22°C and 60% relative humidity, under short day conditions (8 h light/16 h dark). Root inoculations with *R. solanacearum* GM1000 and *ΔpopP2* strains were performed on 3‐week‐old plants. The *arid2/3/4* triple mutant was generated by crossing the single mutants *arid2* (SALK_026835), *arid3* (SALK_022359), and *arid4* (SALK_007400) (Tan et al., 2018).

### Construction of plasmids

Standard DNA cloning methods (Sambrook et al., 1989), PCR, and Gateway technology (Invitrogen) were used for plasmid construction. All primer sequences are listed in Table S2. PCR products flanked by the attB sites were recombined into the pDONR207 vector (Invitrogen) via a BP reaction to create the corresponding entry clones with attL sites (pENTR). The pENTR‐RRS1‐R_Cterm_, pENTR‐PopP2, pENTR‐PopP2‐C321A, pENTR‐Xop6_224‐565_ (pDONR207 vector backbone) clones used in this study have been previously described (Lauber et al., 2024; Le Roux et al., 2015). Full‐length PWWP1 and ARID cDNA clones (ARID1, ARID2, ARID3, and ARID4) and the truncated versions of ARIDs (ARID2_Cterm_, ARID3_Cterm_, ARID4_Cterm_, ARID3_1–92_, ARID3_93–236_, ARID3_1–236_) flanked with the AttB1 and AttB2 recombination sites were amplified from cDNA (Col‐0) using PrimeStar HS DNA polymerase from Takara Bio Inc. (Otsu, Japan). The introduction of the acetyl mimetic mutations in ARID3‐11KQ variant was performed by gene synthesis (the nucleotide sequence of ARID3‐11KQ flanked with AttB1 and AttB2 sites is indicated in Table S2). The pENTR‐PopP2‐3HA and pENTR‐PopP2‐C321A‐3HA were generated by two‐step PCR‐based methods with PopP2‐3HA and PopP2‐C321A‐3HA flanked with *Nco*I and *Avr*II restriction sites. *Nco*I/*Avr*II‐digested fragments were then introduced by ligation in *Nco*I/*Avr*II‐digested pETDuet to produce the pETDuet‐PopP2‐3HA and pETDuet‐PopP2‐C321A‐3HA plasmids used in the bacterial acetylation assay. The pDEST pBIN‐35S‐GWY‐3HA‐Nluc and pBIN‐35S‐GWY‐3Flag‐Cluc vectors used in the SLC assay were obtained by insertion of a blunt‐end chloramphenicol/*ccdB* resistance gateway cassette (Invitrogen) in SnaBI‐digested pBIN‐35S‐3HA‐Nluc and pBIN‐35S‐3Flag‐Cluc (with the Nluc and Cluc moieties corresponding to the residues 1–416 and 398–550 of the firefly luciferase, respectively). The pDEST pB7‐35S‐GWY‐mCherry vector is derived from the pB7FWG2‐35S‐GWY‐eGFP with replacement of the eGFP coding sequence with the sequence of the mCherry using PCR‐based methods. The inserts cloned in pENTR vectors were then recombined in pDEST vectors *via* LR reaction (Invitrogen). All plasmids used for cloning in this study were transformed into *E. coli* strain DH5α using electroporation (0.1 cm cuvette, 1.8 kV pulse, 25 μF and 200 Ω) and selected on LB agar medium with appropriate antibiotics. Plasmid DNAs were extracted with the Wizard DNA plasmid purification kit (Promega) and sequenced using either Sanger‐based sequencers or a MinION NanoSeq sequencer (Oxford Nanopore Technology) with the resulting sequencing data processed thanks to the Long read Plasmid and PCR assembly pipeline NanoSeq release v0.1.3 (Carlier & Moreau, 2024).

### Yeast two‐hybrid assays

Constructs for yeast two‐hybrid analysis were prepared in the MatchMaker GAL4 two‐hybrid system (Clontech) using the pGBG‐GWY (BD fusion) and pGAD‐GWY (AD fusion) vectors to express bait and prey proteins, respectively. All expressed proteins N‐terminally fused with either the BD or AD domain of GAL were tagged with a c‐myc or an HA epitope, respectively. Bait and prey plasmids were introduced in the AH109 yeast strain by using the LiAc/Salmon Sperm carrier DNA/PEG method (Gietz & Schiestl, 2007). Transformed cells were plated on minimal SD−Trp−Leu (SD‐TL) medium and incubated for 3–4 days at 30°C. Transformed yeast cells were incubated in liquid SD‐TL medium under shaking at 30°C overnight. Cell density was checked at OD_600nm_ and adjusted to final OD_600nm_ = 0.5 in sterile ultrapure water. A dilution gradient was performed (from 1 to 10^−3^) to plate the cells on different media: non‐selective (SD−Trp−Leu) and selective (SD−Trp−Leu−His + 5 mm 3‐AT, where 1,2,4‐triazole [3‐AT] serves as competitive inhibitor of *HIS3* gene product), and incubated for 4 to 5 days at 30°C. Protein expression in yeast cells was evaluated following incubation of the different yeast strains under shaking at 30°C overnight in SD‐TL media. For each combination of bait/prey proteins, the cell density of an overnight culture was adjusted to OD_600nm_ = 1. Total yeast proteins were extracted according to a LiAc/NaOH pre‐treatment protocol as previously described (Zhang et al., 2011). Samples were denatured for 3 min at 95°C in Laemmli buffer (2×) supplemented with β‐mercaptoethanol 5% before separation by SDS‐PAGE and immunoblotting with anti‐c‐myc‐HRP and anti‐HA‐HRP conjugated antibodies.

### *Agrobacterium*‐mediated transient expression assay in *N. benthamiana*

For transient expression in *N. benthamiana* leaf epidermal cells, *A. tumefaciens* strains were grown in liquid YEB medium containing appropriate antibiotics for 16 h at 28 °C under shaking. Cells were harvested by centrifugation and resuspended in infiltration medium (10 mm MES pH 5.6, 10 mm MgCl_2_, 150 μm acetosyringone) at OD_600nm_ = 0.25. For co‐expression, bacterial suspensions carrying individual constructs were mixed in a 1:1 ratio (total OD_600nm_ = 0.5). When three different *A. tumefaciens* strains were mixed, a 1:1:1 ratio was used (total OD_600nm_ = 0.75). After incubation at room temperature for 1 h, bacteria were infiltrated into the leaves of 4‐week‐old *N. benthamiana* plants using a needleless syringe. Plants were incubated for 48 h in growth chambers under controlled conditions before harvesting leaf disc samples.

### Confocal microscopy

*Agrobacterium*‐mediated transient expression in *N. benthamiana* was performed using strains carrying the PopP2‐mCherry, ARID2‐eGFP, ARID3‐eGFP, ARID3‐11KQ‐eGFP, and ARID4‐eGFP constructs. Confocal microscopy observations and sampling for immunoblot analysis were conducted at 48 h post‐*Agrobacterium* infiltration in *N. benthamiana* leaves. For protein subcellular localization, GFP and mCherry fluorescence was analyzed with a confocal laser scanning microscope (TCS SP8; Leica) using a ×25 water immersion objective lens (numerical aperture 0.95; HCX PL APO CS2). GFP and mCherry fluorescence were excited with the 488/561 nm ray lines of the lasers and recorded in one of the confocal channels in the 500–550/580‐620 nm emission range, respectively. The images were acquired in the sequential mode using Leica LAS X software (version 3.0). The signal (detector gain) was adjusted on the sample transiently expressing the ARIDx‐eGFP fusion protein alone and then kept unchanged or lower if saturated for the sample co‐expressing also PopP2‐mCherry.

### Split‐luciferase complementation assay

The different Nluc and Cluc fusion proteins were transiently expressed in *N. benthamiana* leaves using *Agrobacterium*‐mediated transformation. For luminescence quantification, single 4 mm punch disks were harvested 48 h later and placed into wells of a 96‐well plate. Samples were washed with 100 μl of ultrapure sterile water for 5 min and then incubated in 100 μl of luciferin substrate (1 mm, XenoLight d‐Luciferin; Perkin Elmer, Waltham, MA, USA) as previously described (Chen et al., 2008; Yu et al., 2020). After 10 min of incubation, light emission was quantified in each well for 5 sec using a luminometer (PerkinElmer; VICTOR Nivo). For each interaction tested, 24 technical replicates were performed (24 leaf disks/plate i.e., eight leaf disks per plant from three plants per biological replicate). Each experiment was replicated at least three times independently. As a positive control, PopP2‐3HA‐Nluc and RRS1‐R_Cterm_‐3Flag‐Cluc proteins were used, based on the previously reported physical interaction between these two proteins (Le Roux et al., 2015).

### Purification and immunoblot analysis of proteins transiently expressed in *N. benthamiana*

For *Agrobacterium*‐mediated transient expression experiments in *N. benthamiana*, protein samples were prepared from four leaf discs (8 mm diameter) harvested 48 h after *Agrobacterium*‐mediated transformation and homogenized in 800 μl of ice cold buffer (50 mm Tris–HCl pH 7.5, 150 mm NaCl, 10 mm EDTA, 2 mm DTT, 1× Plant protease cocktail inhibitor (Sigma), 0.2% Triton). Protein extracts were rotated for 10 min at 4°C and centrifuged at 13 000 ***g*** for 2 min at 4°C to collect the supernatant. Fifty microliters of the protein extract was denatured at 100°C for 3 min in 50 μl of 2 × laemmli buffer (CE, crude extract). For immunoprecipitation of GFP‐tagged proteins, the remaining supernatant was transferred to a new tube and incubated with 8 μl of pre‐equilibrated GFP‐trap agarose beads (Chromotek) for 50 min at 4°C under stirring. Beads were washed three times with 800 μl of ice cold buffer before denaturation in 2 × laemmli buffer at 95°C for 3 min (IP‐GFP).

Protein samples were analyzed by immunoblotting (SDS‐PAGE using 4–15% pre‐casted gels [Bio‐Rad]). After migration, proteins were transferred on a nitrocellulose membrane using the Trans‐Blot Turbo transfer system (Bio‐Rad). Transferred proteins were visualized by Ponceau S red staining (0.5% Ponceau S [w/v], 5% anhydrous acetic acid). PageRuler Prestained Protein Ladder (Thermofischer) was used to estimate protein molecular weights. Membranes were blocked in a 2% milk TBST (Tris Buffer Saline‐Tween‐20; 50 mm Tris–HCl pH 7.5, 150 mm NaCl, 0.1% Tween‐20) solution before immunodetection. The following primary antibodies were used in this study: anti‐c‐myc‐HRP (mouse monoclonal, clone 9E10, dilution 1:10 000; Roche), anti‐HA‐HRP (mouse monoclonal, clone 3F10, dilution 1:5000; Roche), anti‐Flag‐M2‐HRP (mouse monoclonal, dilution 1:5000; Sigma), anti‐GFP (mouse monoclonal, dilution 1:3000; Roche), anti‐Acetylated Lysine (Ac‐K‐103, dilution 1:2000; Cell Signaling Technology), anti‐His6‐HRP (mouse monoclonal, dilution 1:25 000; Roche). The appropriate horseradish peroxidase‐conjugated secondary antibody was applied to the membranes: goat anti‐mouse IgG‐HRP (Bio‐Rad; dilution 1:10 000, for detection of anti‐GFP antibodies); goat anti‐mouse IgG2a‐HRP (Bio‐Rad; dilution 1:5000, for detection of anti‐Ac‐K antibodies). Immunodetections were performed using Clarity Western ECL substrate reagent (Bio‐Rad, Hercules, CA, USA). Chemiluminescence was captured using a G:BOX system (Syngene, Iselin, NJ and Baltimore, MD, USA).

### Bacterial acetylation assay

Single colonies of *E. coli* Rosetta (DE3) cells (Novagen) co‐transformed with pCDF‐GST‐ARID3‐His6 and either pETDuet‐PopP2‐3HA or pETDuet‐PopP2‐C321A‐3HA were grown in 50 ml of LB medium containing carbenicillin (50 μg ml^−1^), spectinomycin (50 μg ml^−1^) and chloramphenicol (30 μg ml^−1^) at 37°C to an OD_600nm_ = 0.6 and induced with 125 μm IPTG (isopropyl‐β‐thiogalactopyranoside) for 4 h 30 min at 28°C. Pelleted cells were concentrated 25 times in ice‐cold protein extraction buffer (50 mm Tris–HCl pH 7.5, 150 mm NaCl, 1 mm phenylmethylsulfonyl fluoride (PMSF; Sigma Aldrich), 1 mm sodium butyrate, 0.1% Triton, 5 mm imidazole) and lysed using a French press. After centrifugation, supernatants were incubated with 100 μl of pre‐equilibrated Ni‐NTA agarose beads (Qiagen) for 2 h at 4°C with rotation. Beads were recovered by centrifugation (5000 ***g*** for 10 min at 4°C) and then washed three times in 10 ml of protein extraction buffer for 10 min at 4°C under stirring. Purified proteins were denatured in laemmli buffer (2×) for 3 min at 95°C.

### Mass spectrometry‐based proteomic analyses

For identification of acetylated residues in ARIDs, protein samples from *N. benthamiana* and from *E. coli* were used. Whole *N. benthamiana* leaves co‐expressing eGFP‐tagged ARID proteins with PopP2‐3HA or PopP2‐C321A‐3HA were collected at 48 hpi. Total proteins were extracted from 3 to 4.5 g of fresh material (FM) ground in liquid nitrogen and resuspended in protein extraction buffer (1 ml for 1 g FM, 50 mm Tris–HCl pH 7.5, 150 mm NaCl, 10 mm EDTA, 2 mm DTT, 1 × plant protease cocktail inhibitor [Sigma], 0.2% Triton, 10 mm sodium butyrate). Total protein extracts were centrifuged and the supernatant (protein extract) was filtered on miracloth. About 50 μl of the protein extract was sampled, mixed in 50 μl of 2 × laemmli buffer and denatured at 95°C for 3 min (CE, crude extract). The remaining protein extract was incubated under shaking at 4°C with pre‐equilibrated GFP‐trap agarose beads (Chromotek GmbH, Planegg, Germany) for 1 h. GFP‐trap agarose beads were collected after centrifugation and the supernatant was discarded. GFP‐trap agarose beads were washed four times in protein extraction buffer. Immunoprecipitated proteins were resuspended in 2 × laemmli buffer and denatured at 95°C for 3 min. Affinity purified proteins (from *N. benthamiana* and Rosetta DE3 cells) were migrated on an SDS‐PAGE gel. After Coomassie blue staining (InstantBlue Coomassie Protein Stain; Abcam Inc., Waltham, MA, USA), gel bands were cut out and proteins were digested in‐gel using trypsin (Promega; sequencing grade) as previously described (Casabona et al., 2013), except that tris(2‐carboxyethyl)phosphine hydrochloride was used instead of dithiothreitol. The resulting peptides were analyzed by online nanoliquid chromatography coupled to MS/MS (Ultimate 3000 RSLCnano and Q‐Exactive HF; Thermo Fisher Scientific). For this purpose, the peptides were sampled on a precolumn (300 μm × 5 mm PepMap C18; Thermo Scientific) and separated in a 75 μm × 250 mm C18 column (Aurora Generation 3, 1.7 μm; IonOpticks) using an acetonitrile gradient for 35 min. The MS and MS/MS data were acquired using Xcalibur 2.9 (Thermo Fisher Scientific, Waltham, MA, USA).

Peptides and proteins were identified by Mascot (version 2.8; Matrix Science) through concomitant searches against the Uniprot database (*N. benthamiana* or *E. coli* BL21‐DE3 taxonomies), a homemade database containing the sequences of recombinant proteins, and a homemade database containing the sequences of classical contaminant proteins found in proteomic analyses (human keratins, trypsin…). Trypsin/P was chosen as the enzyme and two missed cleavages were allowed. Precursor and fragment mass error tolerances were set respectively at 10 and 20 ppm. Peptide modifications allowed during the search were: Carbamidomethyl (C, fixed), Acetyl (K, S, T, variable), Acetyl (Protein N‐term, variable) and Oxidation (M, variable). The Proline software version 2.2.0 (Bouyssié et al., 2020) was used for the compilation, grouping, and filtering of the results (conservation of rank 1 peptides, peptide length ≥6 amino acids, false discovery rate of peptide‐spectrum‐match identifications <1% (Couté et al., 2020), and minimum of one specific peptide per identified protein group). MS data have been deposited to the ProteomeXchange Consortium via the PRIDE partner repository (Perez‐Riverol et al., 2019) with the dataset identifier PXD055620. Proline was then used to perform a spectral counting‐based quantification of the identified protein groups. Only class I acetylated lysines (localization probability ≥0.75) identified in peptide‐spectrum‐matches with Mascot score >30 were further considered.

### *R. solanacearum* inoculation assay

Three‐week‐old *A. thaliana* Col‐0 and *arid2/3/4* plants (*n* ≥ 30 for each line in each condition) were inoculated by soil‐drenching with a *R. solanacearum* bacterial suspension (strains GMI1000 or *ΔpopP2*) containing 10^7^ colony‐forming units per ml (CFU ml^−1^). After a 20‐min incubation in the bacterial solution, plants were transferred to a new tray. Inoculated plants were incubated in a dedicated growth chamber (12 h day/12 h night, 27°C, 75% humidity). Symptoms development was rated daily for 12 days on a scale of 0–4 (disease index [DI]) relative to the percentage of wilted leaves on the aerial part of the plants (0 = 0% wilted, 1 = 25% wilted, 2 = 50% wilted, 3 = 75% wilted, 4 = 100% wilted). To assess survival rates, DI was converted into binary data considering DI ≥ 3 → 1 (death) and DI < 3 → 0 (survival). A Kaplan–Meier survival analysis was then performed on these binary data. For bacteria quantification in Arabidopsis leaves upon *R. solanacearum* inoculation, the aerial parts of Col‐0 and *arid2/3/4* plants inoculated with *R. solanacearum* GMI1000 strain were collected (*n* = 6 for each genotype and for one biological replicate) at DI_mean(Col‐0)_ = 1. Leaf tissues were weighed, decontaminated in 70% ethanol for 3 min, and washed in two consecutive baths of sterile water. Aerial parts were then ground and suspended in sterile water (in a 1 ml:1 g Fresh Material ratio). CFUs were counted by spreading serial dilutions (between 10^−1^ and 10^−5^, with two spreading replicates per sample) on solid SMSA medium (supplemented with 2.5 μg ml^−1^ violet crystal, 12.5 μg ml^−1^ bacitracin, 50 μg ml^−1^ polymyxin B, 24 μg ml^−1^ chloramphenicol, 0.25 μg ml^−1^ penicillin G and 22 U ml^−1^ nystatin) after 36 h of incubation at 28°C.

### Protein sequence alignment

The amino acid sequences of the ARID proteins (ARID2 [786 residues], ARID3 [398 residues] and ARID4 [457 residues]) were considered to perform a multiple sequence alignment using the Scoring Matrix Blosum 62 parameters in Clone Manager 9 Professional Edition software.

### Statistical analysis

For SLC assay‐related measurements, data were analyzed and represented as the mean ± SEM using GraphPad Prism 10.0.3; the horizontal line in the graphs indicates the median. For split‐luciferase‐related data, one‐way anova tests were performed. Stars between two columns indicate statistically significant differences between different tests (****P < 0.0001; ***P < 0.001; ns = non‐significant).

For DI curves, the area under the curves was calculated using the Agricolae package in Rstudio, and statistical analysis was performed using a pairwise Wilcoxon rank test, adjusted with the Benjamini Hochberg method.

For the Kaplan–Meier survival analysis, curves were computed by PRISM, version 10 (GraphPad Software Inc., San Diego, CA, USA) and statistical analysis was performed using the Gehan–Breslow–Wilcoxon method.

For the bacterial growth analysis, plots were computed by PRISM, version 10 (GraphPad Software Inc.), and statistical analysis was performed using a Wilcoxon rank test. For all statistical analyses, the threshold for significance was set for *P*‐value <0.05.

### ACCESSION NUMBERS

*ARID1* (At2g46040), *ARID2* (At2g17410), *ARID3* (At1g20910), *ARID4* (At1g76510), PopP2 (CAD14570), *RRS1‐R* (At5g45260), NbWRK8 (BAI63296.1), *PWWP1* (At3g03140), *arid2/3/4* (SALK_026835/SALK_022359/SALK_007400).

## AUTHOR CONTRIBUTIONS

LM‐W, VP, and LD designed the research. LM‐W, MG, MC, RC, CVi, YM, CVe, YC, VP, and LD performed research. LM‐W, MG, CVe, YC, VP, and LD analyzed the data. LM‐W, VP, and LD wrote the manuscript with inputs from all the authors. All authors read and approved the final manuscript.

## CONFLICT OF INTEREST

The authors declare no conflict of interest.

## Supporting information


**Figure S1.** PopP2 and XopJ6 physically interact with ARID3 C‐terminal part (related to Figure 1).
**Figure S2.** Immunodetection of eGFP‐tagged ARID2, ARID3, and ARID4 expressed alone or with PopP2‐mCherry (related to Figure 2).
**Figure S3.** Immunodetection of different fusion proteins used in the SLC assay (related to Figure 3).
**Figure S4.** Immunodetection of split‐luciferase fusion proteins (related to Figure 4) and Y2H data showing that PopP2‐C321A associates with ARID3.
**Figure S5.** PopP2 associates with PWWP1 in yeast and promotes ARID3/PWWP1 and ARID4/PWWP1 associations (related to Figure 5).
**Figure S6.** *In planta* acetylation of ARID3 and NbWRKY8 in presence of active PopP2.
**Figure S7.** Interacting abilities of the acetyl‐mimetic ARID3‐11KQ variant towards itself, PopP2, and PWWP1.
**Figure S8.** ARID3‐11KQ‐eGFP is nucleo‐cytoplasmic, by contrast to ARID3‐eGFP that specifically accumulates in the plant nucleus.

**Table S1.** List of acetylated K, S, and T residues detected by LC–MS/MS analyses performed on ARID2, ARID3, and ARID4 proteins co‐expressed with either PopP2 or PopP2‐C321A.

**Table S2.** List of bacterial strains, plasmid constructs, and oligonucleotides used in this study.

## Data Availability

All relevant data can be found within the manuscript and its supporting materials.

## References

[tpj70205-bib-0001] Bernoux, M. , Timmers, T. , Jauneau, A. , Brière, C. , de Wit, P. , Marco, Y. et al. (2008) Rd19, an Arabidopsis cysteine protease required for RRS1‐R‐mediated resistance, is relocalized to the nucleus by the *Ralstonia solanacearum* PopP2 effector. The Plant Cell, 20(8), 2252–2264. Available from: 10.1105/tpc.108.058685 18708476 PMC2553607

[tpj70205-bib-0002] Bondino, H.G. , Valle, E.M. & ten Have, A. (2012) Evolution and functional diversification of the small heat shock protein/α‐crystallin family in higher plants. Planta, 235(6), 1299–1313. Available from: 10.1007/s00425-011-1575-9 22210597

[tpj70205-bib-0003] Bouyssié, D. , Hesse, A.‐M. , Mouton‐Barbosa, E. , Rompais, M. , Macron, C. , Carapito, C. et al. (2020) Proline: an efficient and user‐friendly software suite for large‐scale proteomics. Bioinformatics, 36, 3148–3155. 32096818 10.1093/bioinformatics/btaa118 PMC7214047

[tpj70205-bib-0004] Carlier, A. & Moreau, S. (2024) CarlierLab/NanoSeq: NanoSeq v0.1.3 (v0.1.3). *Zenodo*.

[tpj70205-bib-0005] Casabona, M.G. , Vandenbrouck, Y. , Attree, I. & Couté, Y. (2013) Proteomic characterization of *Pseudomonas aeruginosa* PAO1 inner membrane. Proteomics, 13, 2419–2423. 23744604 10.1002/pmic.201200565

[tpj70205-bib-0006] Chakraborty, J. (2021) In‐silico structural analysis of *Pseudomonas syringae* effector HopZ3 reveals ligand binding activity and virulence function. Journal of Plant Research, 134, 599–611. 33730245 10.1007/s10265-021-01274-8

[tpj70205-bib-0007] Chen, H. , Zou, Y. , Shang, Y. , Lin, H. , Wang, Y. , Cai, R. et al. (2008) Firefly luciferase complementation imaging assay for protein‐protein interactions in plants. Plant Physiology, 146, 368–376. 18065554 10.1104/pp.107.111740 PMC2245818

[tpj70205-bib-0057] Chen, X. , Liu, C. , Wang, H. , Liu, Q. , Yue, Y. , Duan, Y. et al. (2024) *Ustilaginoidea virens*‐secreted effector Uv1809 suppresses rice immunity by enhancing OsSRT2‐mediated histone deacetylation. Plant Biotechnology Journal, 22, 148. 37715970 10.1111/pbi.14174 PMC10754013

[tpj70205-bib-0008] Cheong, M.S. , Kirik, A. , Kim, J.‐G. , Frame, K. , Kirik, V. & Mudgett, M.B. (2014) AvrBsT acetylates Arabidopsis ACIP1, a protein that associates with microtubules and is required for immunity. PLoS Pathogens, 10, e1003952. 24586161 10.1371/journal.ppat.1003952 PMC3930583

[tpj70205-bib-0009] Christensen, D.G. , Xie, X. , Basisty, N. , Byrnes, J. , McSweeney, S. , Schilling, B. et al. (2019) Post‐translational protein acetylation: an elegant mechanism for bacteria to dynamically regulate metabolic functions. Frontiers in Microbiology, 10, 1604. Available from: 10.3389/fmicb.2019.01604 31354686 PMC6640162

[tpj70205-bib-0010] Couté, Y. , Bruley, C. & Burger, T. (2020) Beyond target–decoy competition: stable validation of peptide and protein identifications in mass spectrometry‐based discovery proteomics. Analytical Chemistry, 92, 14898–14906. 32970414 10.1021/acs.analchem.0c00328

[tpj70205-bib-0056] Delplace, F. , Huard‐Chauveau, C. , Berthomé, R. & Roby, D. (2022) Network organization of the plant immune system: from pathogen perception to robust defense induction. The Plant Journal, 109, 447–470. 34399442 10.1111/tpj.15462

[tpj70205-bib-0012] Finkemeier, I. , Laxa, M. , Miguet, L. , Howden, A.J.M. & Sweetlove, L.J. (2011) Proteins of diverse function and subcellular location are lysine acetylated in Arabidopsis. Plant Physiology, 155, 1779–1790. 21311031 10.1104/pp.110.171595 PMC3091095

[tpj70205-bib-0013] Galán, J.E. & Waksman, G. (2018) Protein‐injection machines in Bacteria. Cell, 172, 1306–1318. 29522749 10.1016/j.cell.2018.01.034 PMC5849082

[tpj70205-bib-0014] Gietz, R.D. & Schiestl, R.H. (2007) High‐efficiency yeast transformation using the LiAc/SS carrier DNA/PEG method. Nature Protocols, 2, 31–34. 17401334 10.1038/nprot.2007.13

[tpj70205-bib-0015] Godwin, J. , Govindasamy, M. , Nedounsejian, K. , March, E. , Halton, R. , Bourbousse, C. et al. (2024) The UBP5 histone H2A deubiquitinase counteracts PRCs‐mediated repression to regulate Arabidopsis development. Nature Communications, 15, 667. 10.1038/s41467-023-44546-8 PMC10803359 38253560

[tpj70205-bib-0016] Groenen, P.J. , Merck, K.B. , de Jong, W.W. & Bloemendal, H. (1994) Structure and modifications of the junior chaperone alpha‐crystallin. From lens transparency to molecular pathology. European Journal of Biochemistry, 225, 1–19. 7925426 10.1111/j.1432-1033.1994.00001.x

[tpj70205-bib-0017] Harris, W. , Kim, S. , Völz, R. & Lee, Y. (2023) Nuclear effectors of plant pathogens: distinct strategies to be one step ahead. Molecular Plant Pathology, 24, 637–650. 36942744 10.1111/mpp.13315 PMC10189769

[tpj70205-bib-0018] Herrscher, R.F. , Kaplan, M.H. , Lelsz, D.L. , Das, C. , Scheuermann, R. & Tucker, P.W. (1995) The immunoglobulin heavy‐chain matrix‐associating regions are bound by Bright: a B cell‐specific trans‐activator that describes a new DNA‐binding protein family. Genes & Development, 9, 3067–3082. 8543152 10.1101/gad.9.24.3067

[tpj70205-bib-0019] Jeleńska, J. , Lee, J. , Manning, A.J. , Wolfgeher, D.J. , Ahn, Y. , Walters‐Marrah, G. et al. (2021) *Pseudomonas syringae* effector HopZ3 suppresses the bacterial AvrPto1–tomato PTO immune complex *via* acetylation. PLoS Pathogens, 17, e1010017. 34724007 10.1371/journal.ppat.1010017 PMC8584673

[tpj70205-bib-0020] Jiang, S. , Yao, J. , Ma, K.‐W. , Zhou, H. , Song, J. , He, S.Y. et al. (2013) Bacterial effector activates jasmonate signaling by directly targeting JAZ transcriptional repressors. PLoS Pathogens, 9, e1003715. 24204266 10.1371/journal.ppat.1003715 PMC3814404

[tpj70205-bib-0021] Kai, K. (2023) The phc quorum‐sensing system in *Ralstonia solanacearum* species complex. Annual Review of Microbiology, 77, 213–231. Available from: 10.1146/annurev-micro-032521-030537 37100406

[tpj70205-bib-0022] Kong, L. , Qiu, X. , Kang, J. , Wang, Y. , Chen, H. , Huang, J. et al. (2017) A *Phytophthora* effector manipulates host histone acetylation and reprograms defense gene expression to promote infection. Current Biology, 27, 981–991. 28318979 10.1016/j.cub.2017.02.044

[tpj70205-bib-0023] Korn, S.M. & Schlundt, A. (2022) Structures and nucleic acid‐binding preferences of the eukaryotic ARID domain. Biological Chemistry, 403, 731–747. 35119801 10.1515/hsz-2021-0404

[tpj70205-bib-0024] Landry, D. , González‐Fuente, M. , Deslandes, L. & Peeters, N. (2020) The large, diverse, and robust arsenal of *Ralstonia solanacearum* type III effectors and their in planta functions. Molecular Plant Pathology, 21, 1377–1388. 32770627 10.1111/mpp.12977 PMC7488467

[tpj70205-bib-0025] Lauber, E. , González‐Fuente, M. , Escouboué, M. , Vicédo, C. , Luneau, J.S. , Pouzet, C. et al. (2024) Bacterial host adaptation through sequence and structural variations of a single type III effector gene. iScience, 27, 109224. 38439954 10.1016/j.isci.2024.109224 PMC10909901

[tpj70205-bib-0026] Le Roux, C. , Huet, G. , Jauneau, A. et al. (2015) A receptor pair with an integrated decoy converts pathogen disabling of transcription factors to immunity. Cell, 161, 1074–1088. 26000483 10.1016/j.cell.2015.04.025

[tpj70205-bib-0027] Lee, A.H.‐Y. , Hurley, B. , Felsensteiner, C. et al. (2012) A bacterial acetyltransferase destroys plant microtubule networks and blocks secretion. PLoS Pathogens, 8, e1002523. 22319451 10.1371/journal.ppat.1002523 PMC3271077

[tpj70205-bib-0028] Lee, J. , Manning, A.J. , Wolfgeher, D. , Jelenska, J. , Cavanaugh, K.A. , Xu, H. et al. (2015) Acetylation of an NB‐LRR plant immune‐effector complex suppresses immunity. Cell Reports, 13, 1670–1682. 26586425 10.1016/j.celrep.2015.10.029 PMC4967551

[tpj70205-bib-0029] Lewis, J.D. , Lee, A.H.‐Y. , Hassan, J.A. , Wan, J. , Hurley, B. , Jhingree, J.R. et al. (2013) The Arabidopsis ZED1 pseudokinase is required for ZAR1‐mediated immunity induced by the *Pseudomonas syringae* type III effector HopZ1a. Proceedings of the National Academy of Sciences of the United States of America, 110, 18722–18727. 24170858 10.1073/pnas.1315520110 PMC3831984

[tpj70205-bib-0030] Lewis, J.D. , Wu, R. , Guttman, D.S. & Desveaux, D. (2010) Allele‐specific virulence attenuation of the *Pseudomonas syringae* HopZ1a type III effector *via* the Arabidopsis ZAR1 resistance protein. PLoS Genetics, 6, e1000894. 20368970 10.1371/journal.pgen.1000894 PMC2848558

[tpj70205-bib-0031] Li, H. , Wang, H. , Jing, M. et al. (2018) A *Phytophthora* effector recruits a host cytoplasmic transacetylase into nuclear speckles to enhance plant susceptibility. eLife, 7, e40039. 30346270 10.7554/eLife.40039 PMC6249003

[tpj70205-bib-0032] Li, J. , Xiang, C.‐Y. , Yang, J. , Chen, J.‐P. & Zhang, H.‐M. (2015) Interaction of HSP20 with a viral RdRp changes its sub‐cellular localization and distribution pattern in plants. Scientific Reports, 5, 14016. 26359114 10.1038/srep14016 PMC4642574

[tpj70205-bib-0033] Ma, K.‐W. & Ma, W. (2016) YopJ family effectors promote bacterial infection through a unique acetyltransferase activity. Microbiology and Molecular Biology Reviews, 80, 1011–1027. 27784797 10.1128/MMBR.00032-16 PMC5116873

[tpj70205-bib-0034] Macho, A.P. , Guidot, A. , Barberis, P. , Beuzón, C.R. & Genin, S. (2010) A competitive index assay identifies several *Ralstonia solanacearum* type III effector mutant strains with reduced fitness in host plants. Molecular Plant‐Microbe Interactions, 23, 1197–1205. 20687809 10.1094/MPMI-23-9-1197

[tpj70205-bib-0036] Ngou, B.P.M. , Ding, P. & Jones, J.D.G. (2021) Channeling plant immunity. Cell, 184, 3358–3360. 34171318 10.1016/j.cell.2021.05.035

[tpj70205-bib-0037] Nixon, J.C. , Rajaiya, J. & Webb, C.F. (2004) Mutations in the DNA‐binding domain of the transcription factor Bright act as dominant negative proteins and interfere with immunoglobulin transactivation. Journal of Biological Chemistry, 279, 52465–52472. 15456761 10.1074/jbc.M403028200

[tpj70205-bib-0038] Park, C.‐J. & Seo, Y.‐S. (2015) Heat shock proteins: a review of the molecular chaperones for plant immunity. Plant Pathology Journal, 31, 323–333. 26676169 10.5423/PPJ.RW.08.2015.0150 PMC4677741

[tpj70205-bib-0039] Perez‐Riverol, Y. , Csordas, A. , Bai, J. , Bernal‐Llinares, M. , Hewapathirana, S. , Kundu, D.J. et al. (2019) The PRIDE database and related tools and resources in 2019: improving support for quantification data. Nucleic Acids Research, 47, D442–D450. 30395289 10.1093/nar/gky1106 PMC6323896

[tpj70205-bib-0040] Sambrook, A.J. , Frisch, E. & Maniatis, T. (Eds.). (1989) Molecular cloning: a laboratory manual, 2nd edition. New York: Cold Spring Harbor Laboratory Press.

[tpj70205-bib-0041] Sarris, P.F. , Duxbury, Z. , Huh, S.U. , Ma, Y. , Segonzac, C. , Sklenar, J. et al. (2015) A plant immune receptor detects pathogen effectors that target WRKY transcription factors. Cell, 161, 1089–1100. 26000484 10.1016/j.cell.2015.04.024

[tpj70205-bib-0042] Scharf, K.‐D. , Siddique, M. & Vierling, E. (2001) The expanding family of *Arabidopsis thaliana* small heat stress proteins and a new family of proteins containing α‐crystallin domains (Acd proteins). Cell Stress & Chaperones, 6, 225–237. 11599564 10.1379/1466-1268(2001)006<0225:tefoat>2.0.co;2 PMC434404

[tpj70205-bib-0060] Schator, D. , Gomez‐Valero, L. , Buchrieser, C. & Rolando, M. (2021) Patho‐epigenetics: histone deacetylases as targets of pathogens and therapeutics. microLife, 2, uqab013. 37223249 10.1093/femsml/uqab013 PMC10117728

[tpj70205-bib-0043] Tan, L. , Zhang, C. , Hou, X. , Shao, C.‐.R. , Lu, Y.‐.J. , Zhou, J.‐.X. et al. (2018) The PEAT protein complexes are required for histone deacetylation and heterochromatin silencing. The EMBO Journal, 37, e98770‐19. Available from: 10.15252/embj.201798770 30104406 PMC6166130

[tpj70205-bib-0044] Tasset, C. , Bernoux, M. , Jauneau, A. , Pouzet, C. , Brière, C. , Kieffer‐Jacquinod, S. et al. (2010) Autoacetylation of the Ralstonia solanacearum effector PopP2 targets a lysine residue essential for RRS1‐R‐mediated immunity in Arabidopsis. PLoS Pathogens, 6, e1001202. 21124938 10.1371/journal.ppat.1001202 PMC2987829

[tpj70205-bib-0045] Vailleau, F. & Genin, S. (2023) *Ralstonia solanacearum*: an arsenal of virulence strategies and prospects for resistance. Annual Review of Phytopathology, 61, 25–47. 10.1146/annurev-phyto-021622-104551 37506349

[tpj70205-bib-0046] Vijayapalani, P. , Hewezi, T. , Pontvianne, F. & Baum, T.J. (2018) An effector from the cyst nematode *Heterodera schachtii* derepresses host rRNA genes by altering histone acetylation. The Plant Cell, 30, 2795–2812. 30333146 10.1105/tpc.18.00570 PMC6305986

[tpj70205-bib-0047] Wang, Y. , Pruitt, R.N. , Nürnberger, T. & Wang, Y. (2022) Evasion of plant immunity by microbial pathogens. Nature Reviews. Microbiology, 20, 449–464. 35296800 10.1038/s41579-022-00710-3

[tpj70205-bib-0048] Waters, E.R. & Vierling, E. (2020) Plant small heat shock proteins – evolutionary and functional diversity. New Phytologist, 227, 24–37. 32297991 10.1111/nph.16536

[tpj70205-bib-0049] Williams, S.J. , Sohn, K.H. , Wan, L. , Bernoux, M. , Sarris, P.F. , Segonzac, C. et al. (2014) Structural basis for assembly and function of a heterodimeric plant immune receptor. Science, 344, 299–303. 24744375 10.1126/science.1247357

[tpj70205-bib-0059] Wilsker, D. , Probst, L. , Wain, H.M. , Maltais, L. , Tucker, P.W. & Moran, E. (2005) Nomenclature of the ARID family of DNA‐binding proteins. Genomics, 86, 242–251. 15922553 10.1016/j.ygeno.2005.03.013

[tpj70205-bib-0050] Xing, S. & Poirier, Y. (2012) The protein acetylome and the regulation of metabolism. Trends in Plant Science, 17, 423–430. 22503580 10.1016/j.tplants.2012.03.008

[tpj70205-bib-0051] Xue, H. , Lozano‐Durán, R. & Macho, A.P. (2020) Insights into the root invasion by the plant pathogenic bacterium *Ralstonia solanacearum* . Plants, 9, 516. 32316375 10.3390/plants9040516 PMC7238422

[tpj70205-bib-0052] Yu, G. , Xian, L. , Xue, H. , Yu, W. , Rufian, J.S. , Sang, Y. et al. (2020) A bacterial effector protein prevents MAPK‐mediated phosphorylation of SGT1 to suppress plant immunity. PLoS Pathogens, 16, e1008933. 32976518 10.1371/journal.ppat.1008933 PMC7540872

[tpj70205-bib-0053] Yuan, M. , Ngou, B.P.M. , Ding, P. & Xin, X.‐F. (2021) PTI‐ETI crosstalk: an integrative view of plant immunity. Current Opinion in Plant Biology, 62, 102030. 33684883 10.1016/j.pbi.2021.102030

[tpj70205-bib-0061] Zhang, T. , Lei, J. , Yang, H. , Xu, K. , Wang, R. & Zhang, Z. (2011) An improved method for whole protein extraction from yeast *Saccharomyces cerevisiae* . Yeast, 28, 795–798. 21972073 10.1002/yea.1905

[tpj70205-bib-0054] Zheng, S.‐Y. , Guan, B.‐B. , Yuan, D.‐Y. , Zhao, Q.Q. , Ge, W. , Tan, L.M. et al. (2023) Dual roles of the Arabidopsis PEAT complex in histone H2A deubiquitination and H4K5 acetylation. Molecular Plant, 16, 1847–1865. 37822080 10.1016/j.molp.2023.10.006

[tpj70205-bib-0055] Zhu, H. , Chen, T. , Zhu, M. , Fang, Q. , Kang, H. , Hong, Z. et al. (2008) A novel ARID DNA‐binding protein interacts with SymRK and is expressed during early nodule development in *Lotus japonicus* . Plant Physiology, 148, 337–347. 18633121 10.1104/pp.108.119164 PMC2528112

