# Supplementary material for: Three ARID proteins involved in chromatin remodeling PEAT complexes are targeted by the Ralstonia solanacearum effector PopP2 and contribute to bacterial wilt disease
Source: Plant J. 2025 May 13;122(3):e70205. doi: 10.1111/tpj.70205 (PMC12071340; doi:10.1111/tpj.70205)
Supplement: Supplementary file 1 — Figure S1. PopP2 and XopJ6 physically interact with ARID3 C‐terminal part (related to Figure 1). Figure S2. Immunodetection of eGFP‐tagged ARID2, ARID3, and ARID4 expressed alone or with PopP2‐mCherry (related to Figure 2). Figure S3. Immunodetection of different fusion proteins used in the SLC assay (related to Figure 3). Figure S4. Immunodetection of split‐luciferase fusion proteins (related to Figure 4) and Y2H data showing that PopP2‐C321A associates with ARID3. Figure S5. PopP2 associates with PWWP1 in yeast and promotes ARID3/PWWP1 and ARID4/PWWP1 associations (related to Figure 5). Figure S6. In planta acetylation of ARID3 and NbWRKY8 in presence of active PopP2. Figure S7. Interacting abilities of the acetyl‐mimetic ARID3‐11KQ variant towards itself, PopP2, and PWWP1. Figure S8. ARID3‐11KQ‐eGFP is nucleo‐cytoplasmic, by contrast to ARID3‐eGFP that specifically accumulates in the plant nucleus. [file TPJ-122-0-s002.pdf]

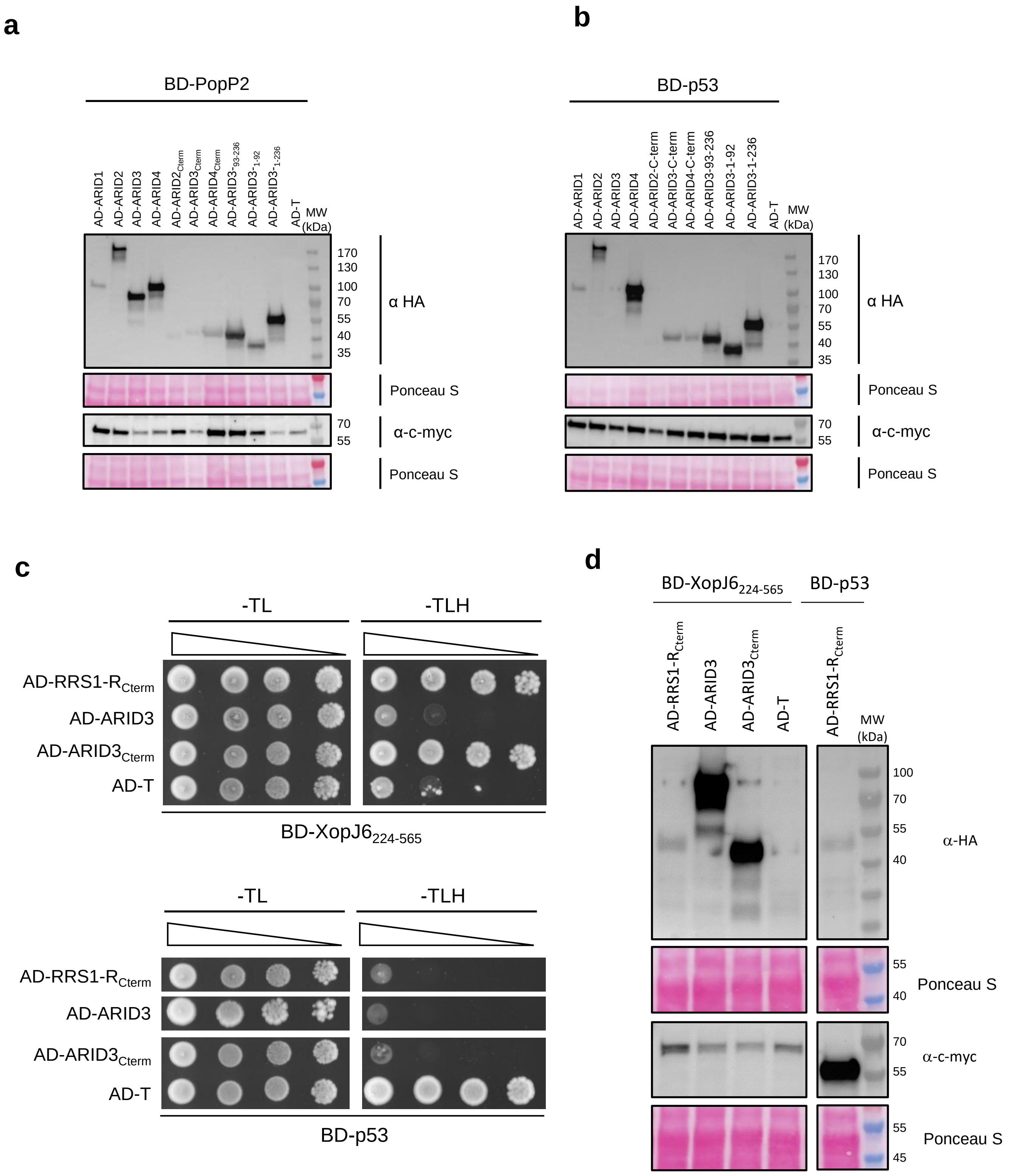

Figure S1. PopP2 and XopJ6 physically interact with ARID3 C-terminal part (related to Figure1)
(a) and (b) Total yeast protein extracts were separated by SDS-PAGE and immunoblot analysis was performed using either anti-c-myc-HRP (for BD-fusion proteins) or anti-HA-HRP (for AD-fusion proteins) antibodies. Ponceau S staining indicates similar amounts of protein loaded between the different lanes.
(c) Yeast two-hybrid (Y2H) assays between XopJ6 catalytic unit (residues 224 to 565) fused to the Gal4 DNA-binding domain (BD-XopJ6$_{224-565}$) and either full-length or truncated ARID3 proteins fused to the GAL4 activation domain. Serial dilutions of transformed yeast cells were grown on selective media (SD/- Trp/-Leu (-TL) or SD/-Trp/-Leu/-His (-TLH)). Plates were incubated at 28°C and photographed five days later. The experiment was repeated three times with similar results. (d) Immunoblot analysis of the various bait and prey proteins expressed in (c).

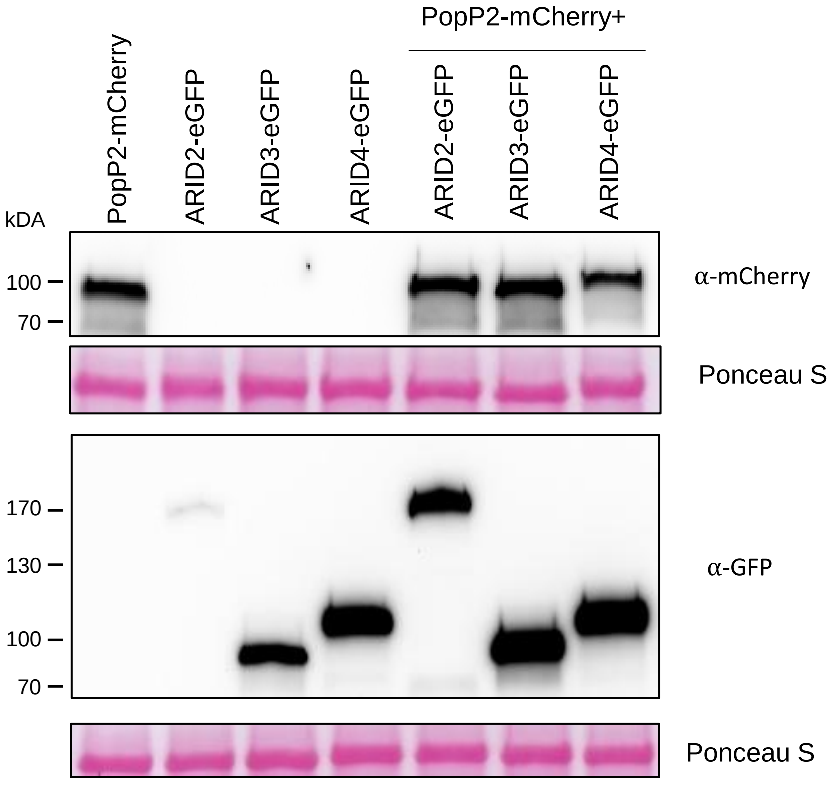

Figure S2. Immunodetection of eGFP-tagged ARID2, ARID3 and ARID4 expressed alone or with PopP2-mCherry (related to Figure 2).

ARID2-eGFP, ARID3-eGFP and ARID4-eGFP were transiently expressed either alone or with PopP2-mCherry in *N. benthamiana* leaves. Samples were harvested at 48h post agro-infiltration. Crude protein extracts (CE) were immunoblotted with anti-mCherry (α-mCherry) and anti-GFP (α-GFP) antibodies. Ponceau S staining of total proteins indicates similar amounts of protein loaded between the different lanes.

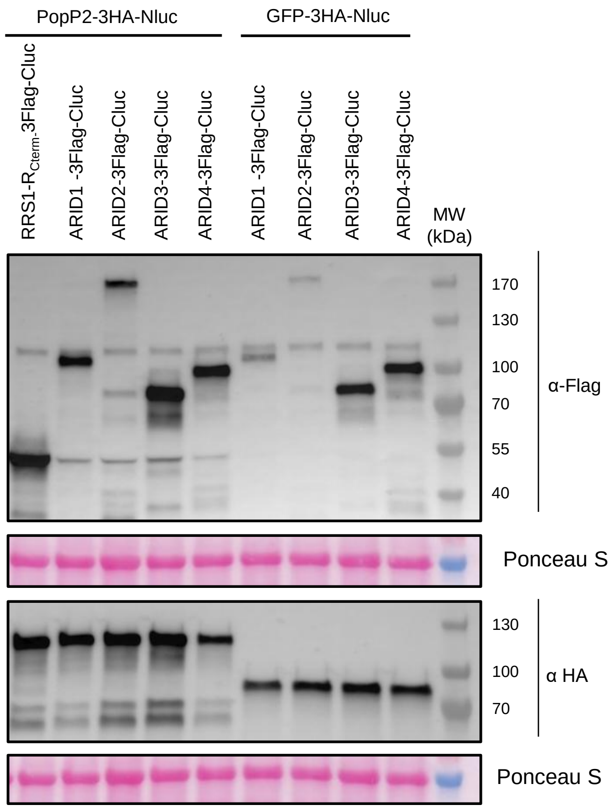

Figure S3. Immunodetection of different fusion proteins used in the SLC assay (related to Figure 3). ARID1/2/3/4 and RRS1-R_{Cterm} C-terminally tagged with a 3Flag epitope, fused to the C-terminal region of the luciferase were transiently co-expressed with 3HA-tagged PopP2 or GFP fused to the luciferase N-terminal region in *N. benthamiana* leaves. Samples were harvested at 48h post agro-infiltration. The different proteins used in the split-luciferase assay were immunodetected using anti-Flag-HRP (for 3Flag-Cluc-fusion proteins) or anti-HA-HRP (for 3HA-Nluc-fusion proteins) antibodies. Ponceau S staining indicates similar amounts of protein loaded between the different lanes.

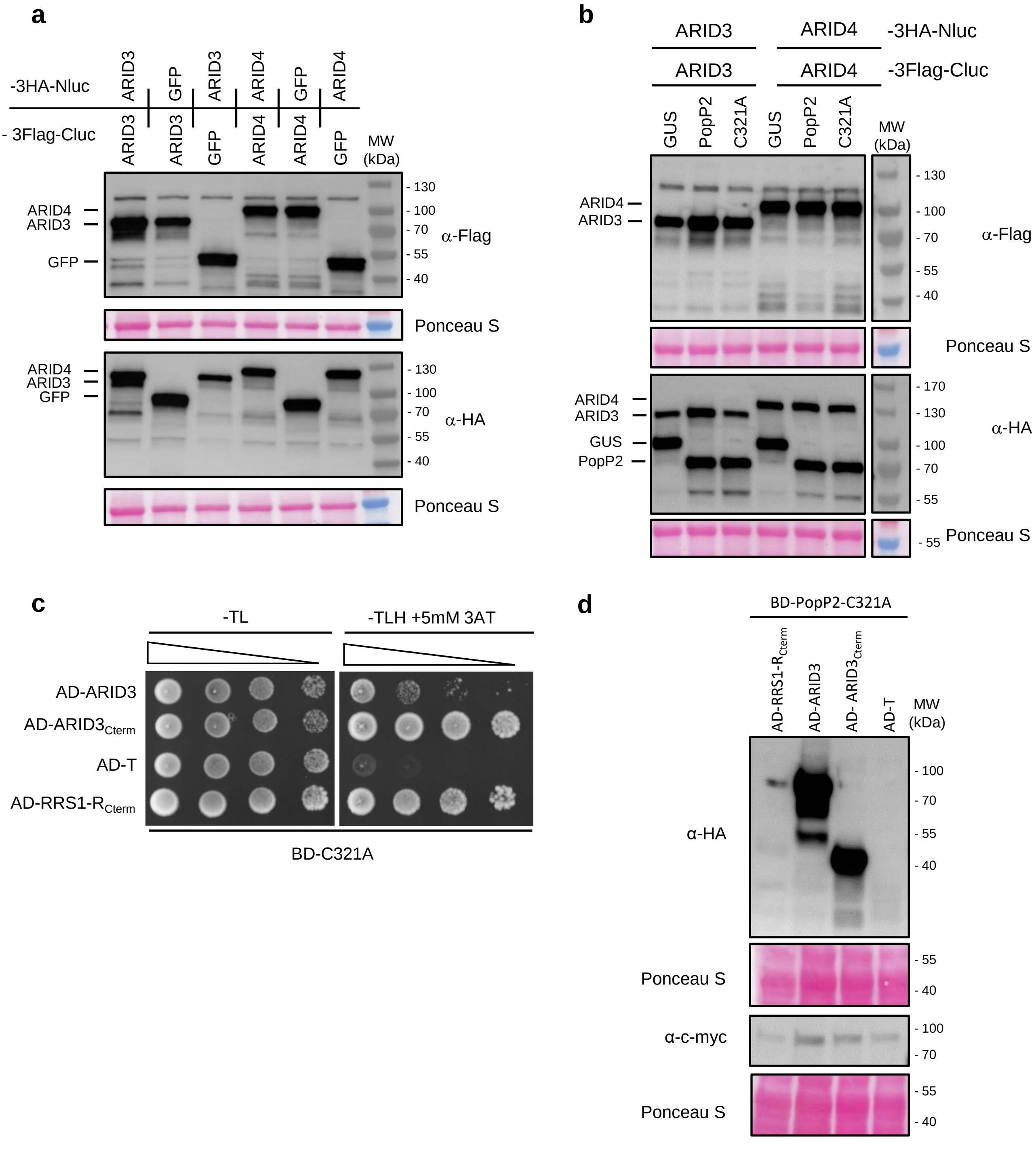

Figure S4. Immunodetection of split-luciferase fusion proteins (related to Figure 4) and Y2H data showing that PopP2-C321A interacts with ARID3.

(a) and (b) Fusion proteins used in the SLC assay related to Figure 4A and 4B were immunodetected using either anti-Flag and anti-HA antibodies. Ponceau S staining of total proteins indicates similar amounts of protein loaded between the different lanes. (c) and (d) The catalytic mutant PopP2-C321A interacts with ARID3 and $ARID3_{Cterm}$ in yeast. ARID3, $ARID3_{Cterm}$ and the positive control $RRS1\text{-}R_{Cterm}$ were N-terminally fused with the Gal4 activation domain and co-expressed as preys in yeast cells with the bait protein PopP2-C321A N-terminally fused with the Gal4 DNA binding domain (BD-C321A). The SV40 large T-antigen (AD-T) construct was used as negative control of protein-protein interaction. Serial dilutions of transformed yeast cells were plated on selective media (SD/-Trp/-Leu (-TL) or SD/-Trp/-Leu/-His (-TLH + 5mM 3AT)). Plates were photographed after five days of incubation at 28°C. The experiment was repeated three times with similar results. Expression of the bait and prey proteins was checked by immunoblot analysis performed on total yeast protein extracts using anti-c-myc-HRP (for BD-fusion proteins) or anti-HA-HRP (for AD-fusion proteins) antibodies. Ponceau S staining indicates the amount of protein loaded between the different lanes.

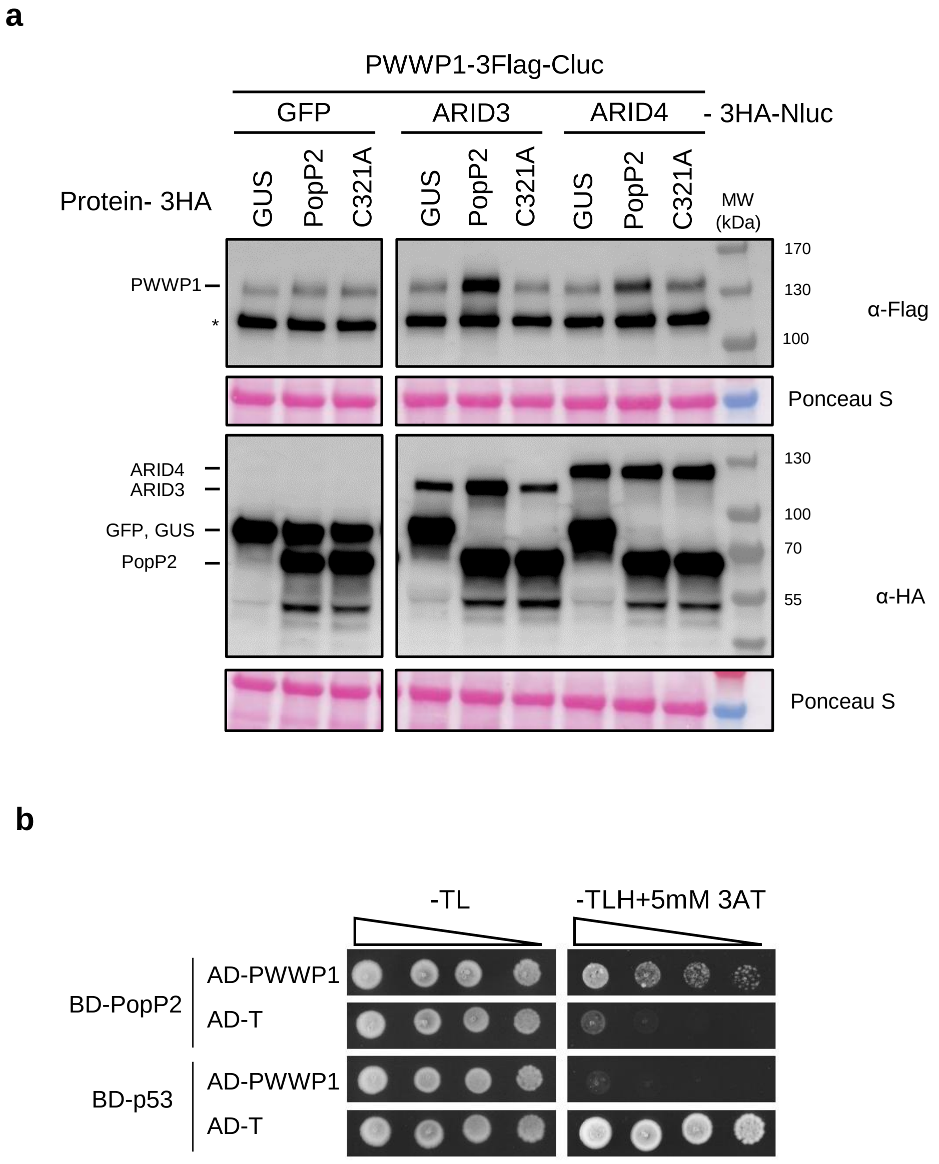

Figure S5. PopP2 associates with PWWP1 in yeast and promotes ARID3/PWWP1 and ARID4/PWWP1 associations (related to Figure 5).

(a) Immunodetection of the fusion proteins used in the Split-luciferase assay was performed using either anti-Flag (for 3Flag-Cluc-fusion proteins) or anti-HA (for 3HA-Nluc-fusion proteins and for GUS/PopP2/C321A) antibodies. Membranes were stained with Ponceau S to assess protein loading. (b) PWWP1 binds PopP2 in yeast cells. In this Y2H assay, PWWP1 was N-terminally fused with the Gal4 activation domain (AD) and co-expressed as prey in yeast cells with BD-PopP2. The murine p53 protein was used as a negative control (BD-p53) for interaction with AD-fusion proteins and as a positive control for interaction with the SV40 large T-antigen (AD-T). Serial dilution of transformed yeast cells were plated on selective media (SD/-Trp/-Leu (-TL) or SD/-Trp/-Leu/-His (-TLH + 5mM 3AT)). Plates were photographed after five days of incubation at 28°C. This experiment was repeated two times with similar results.

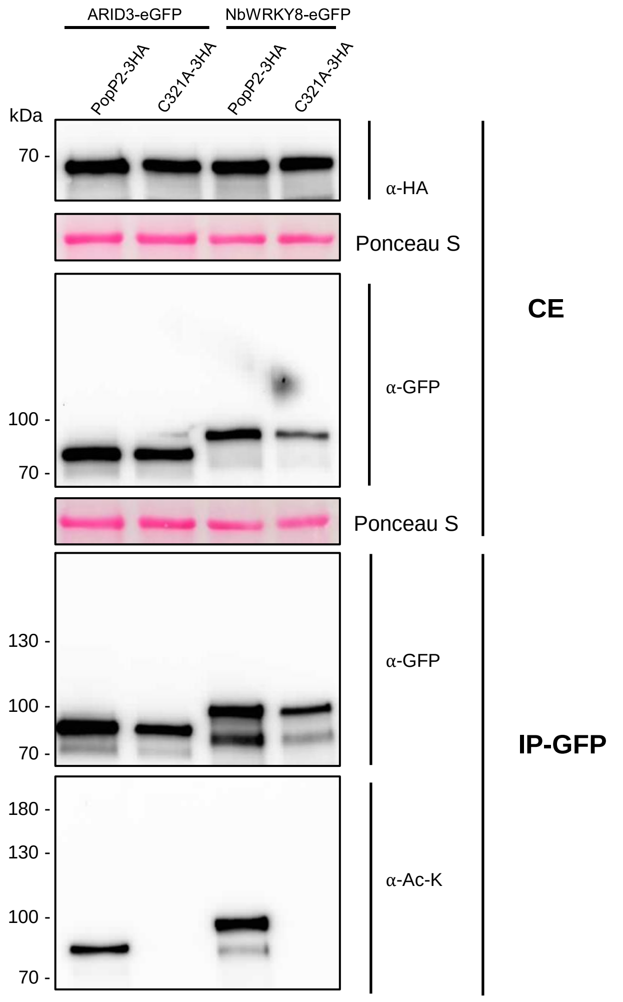

Figure S6. ***In planta*** **acetylation of ARID3 and NbWRKY8 in presence of active PopP2.**
ARID3-eGFP and NbWRKY8-eGFP were transiently expressed with 3HA-tagged active PopP2 or C321A catalytic mutant in N. benthamiana leaves. Samples were harvested at 48hpi. Protein extracts (CE) were immunoblotted with anti-HA (α-HA) and anti-GFP (α-GFP) antibodies. ARID3-eGFP and NbWRKY8-eGFP were immunoprecipitated on GFP-trap agarose beads (IP-GFP) and analysed by immunoblotting with anti-GFP (α-GFP) and anti-acetylated-lysine (α-Ac-K) antibodies. Ponceau S staining indicates the amount of protein loaded in the different lanes.

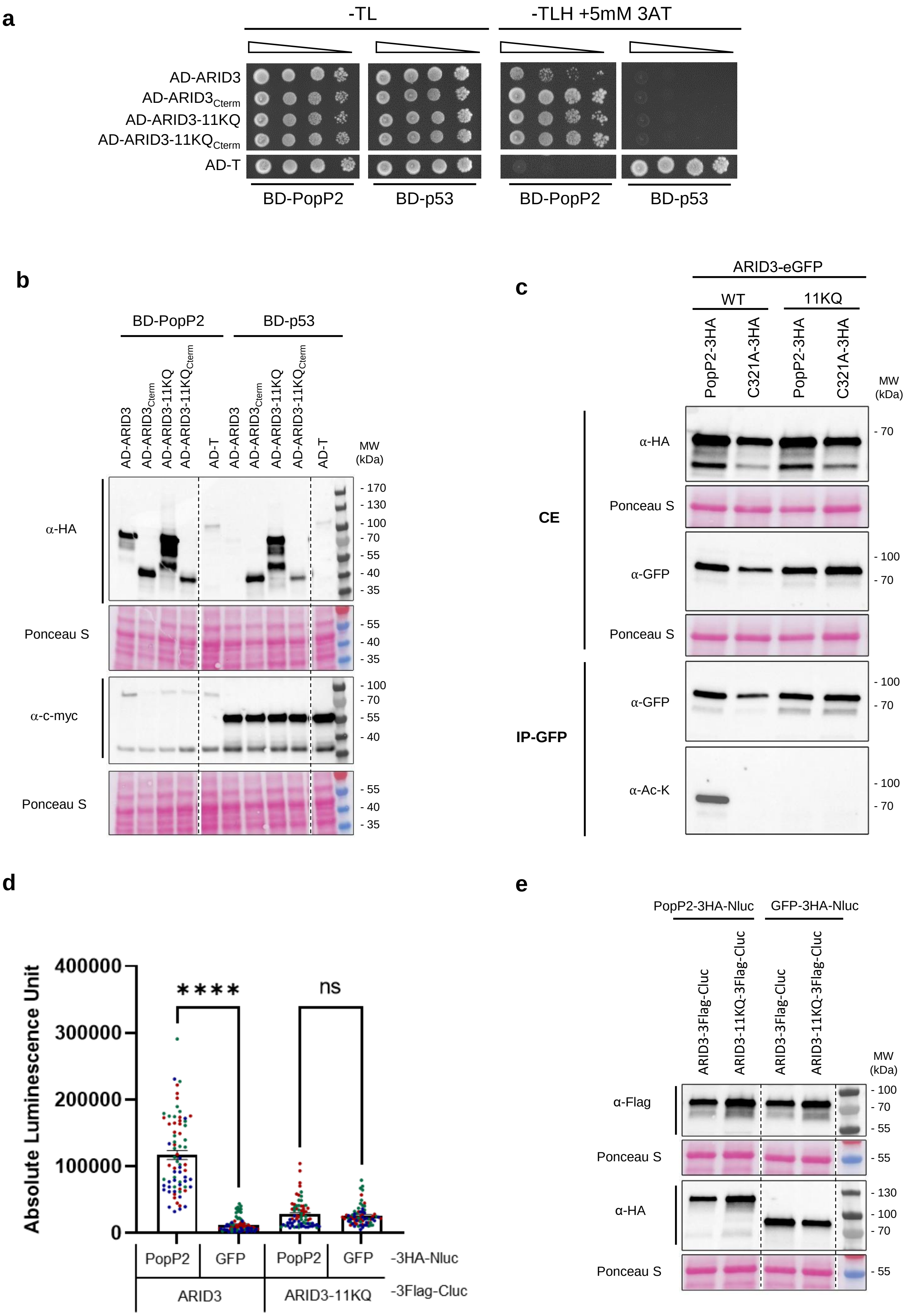

a
-TL
-TLH +5mM 3AT
AD-ARID3
AD-ARID3Cterm
AD-ARID3-11KQ
AD-ARID3-11KQCterm
AD-T
BD-PopP2
BD-p53
BD-PopP2
BD-p53
b
BD-PopP2
BD-p53
AD-ARID3
AD-ARID3Cterm
AD-ARID3-11KQ
AD-ARID3-11KQCterm
AD-T
AD-ARID3
AD-ARID3Cterm
AD-ARID3-11KQ
AD-ARID3-11KQCterm
AD-T
MW (kDa)
α-HA
- 170
- 130
- 100
- 70
- 55
- 40
- 35
Ponceau S
- 55
- 40
- 35
α-c-myc
- 100
- 70
- 55
- 40
Ponceau S
- 55
- 40
- 35
c
ARID3-eGFP
WT
11KQ
PopP2-3HA
C321A-3HA
PopP2-3HA
C321A-3HA
MW (kDa)
CE
α-HA
- 70
Ponceau S
α-GFP
- 100
- 70
Ponceau S
IP-GFP
α-GFP
- 100
- 70
α-Ac-K
- 100
- 70
d
Absolute Luminescence Unit
400000
300000
200000
100000
0
****
ns
PopP2
GFP
PopP2
GFP
-3HA-Nluc
ARID3
ARID3-11KQ
-3Flag-Cluc
e
PopP2-3HA-Nluc
GFP-3HA-Nluc
ARID3-3Flag-Cluc
ARID3-11KQ-3Flag-Cluc
ARID3-3Flag-Cluc
ARID3-11KQ-3Flag-Cluc
MW (kDa)
α-Flag
- 100
- 70
- 55
Ponceau S
- 55
α-HA
- 130
- 100
- 70
Ponceau S
- 55

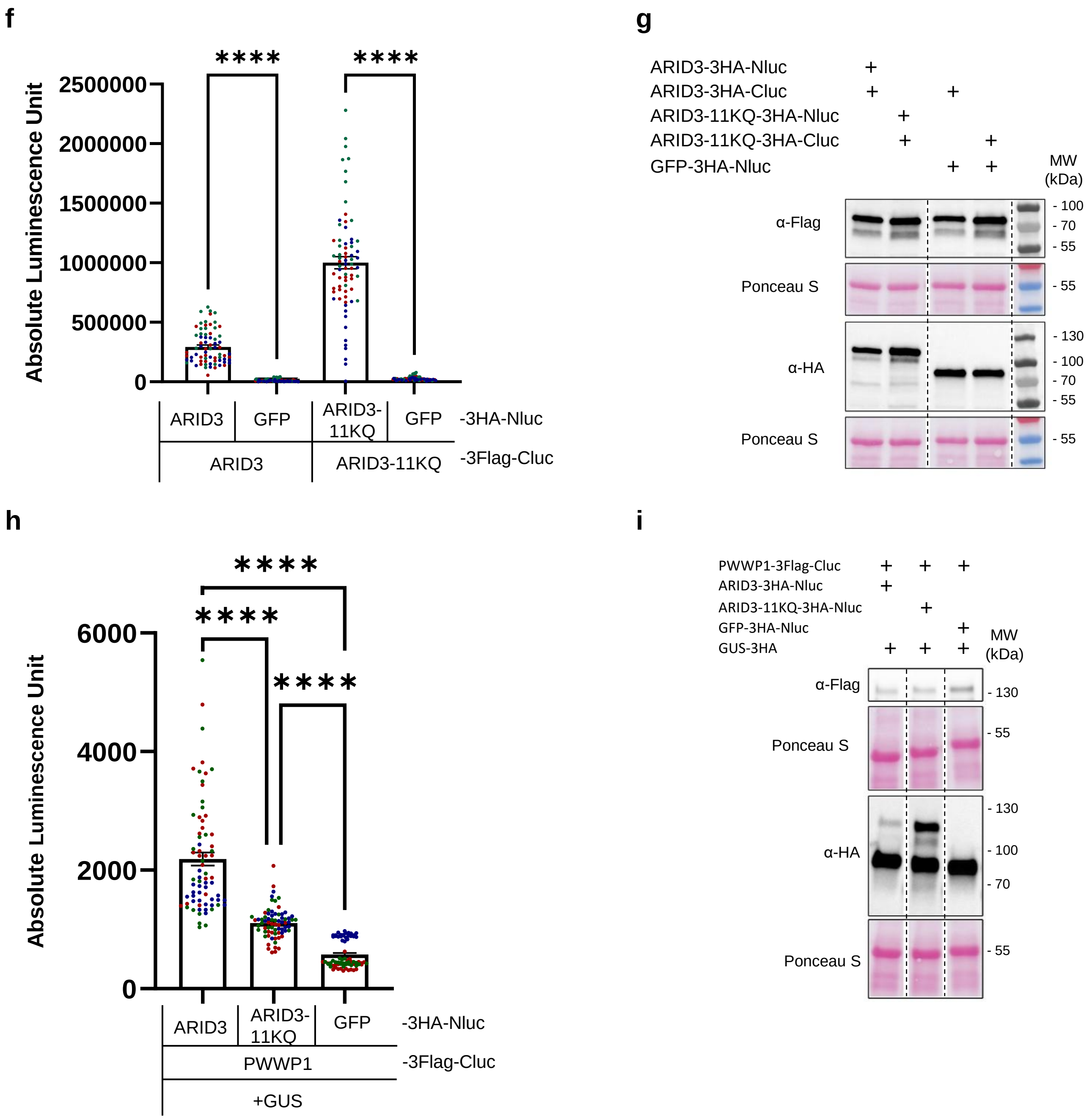

Figure S7. Interacting abilities of the acetyl-mimetic ARID3-KQ variant towards itself, PopP2 and PWWP1.

(a) Yeast two-hybrid (Y2H) assay showing that full-length ARID3-11KQ and its C-terminal part can physically interact with PopP2. (b) Total yeast protein extracts were separated by SDS-PAGE and immunoblot analysis was performed using either anti-c-myc-HRP (for BD-fusion proteins) or anti-HA-HRP (for AD-fusion proteins) antibodies. Ponceau S staining indicates similar amounts of protein loaded between the different lanes. Dashed line indicates that unnecessary lanes were removed. (c) ARID3-11KQ-eGFP is not acetylated in presence of active PopP2 in *N. benthamiana* leaves (see also legend in Figure 6). (d) Quantitative measurement of luciferase-derived signals shows that the interaction between PopP2 and ARID3-11KQ is impaired *in planta* (see also legend in Figure 3). For this Split Luciferase complementation (SLC) assay, the co-expression of GFP-3HA-Nluc with ARID3-3Flag-Cluc or ARID3-11KQ-3Flag-Cluc serve as negative controls. (f) ARID3-11KQ self-associates in living *N. benthamiana* cells. (h) The ability of PWWP1 to interact with ARID3 is negatively impacted by the introduction of the acetyl mimetic substitions in ARID3. For (d)-(f)-(h), leaf samples were collected 48h post agro-infiltration. Bars indicate mean absolute luminescence unit ± s.e.m. Three independent replicates were performed (for each replicate, n=24). Data points displayed on the graph are colored depending on the replicate they were collected from. Statistical analysis was performed using one-way ANOVA with Tukey's test. ****p<0.0001; ns, non-significant. The expression of the different fusion proteins in *N. benthamiana* was checked by immunoblot analysis. For (e)-(g)-(i), dashed lines indicate that the original image was cut to remove unnecessary lanes. Immunoblots are representative of three independent experiments.

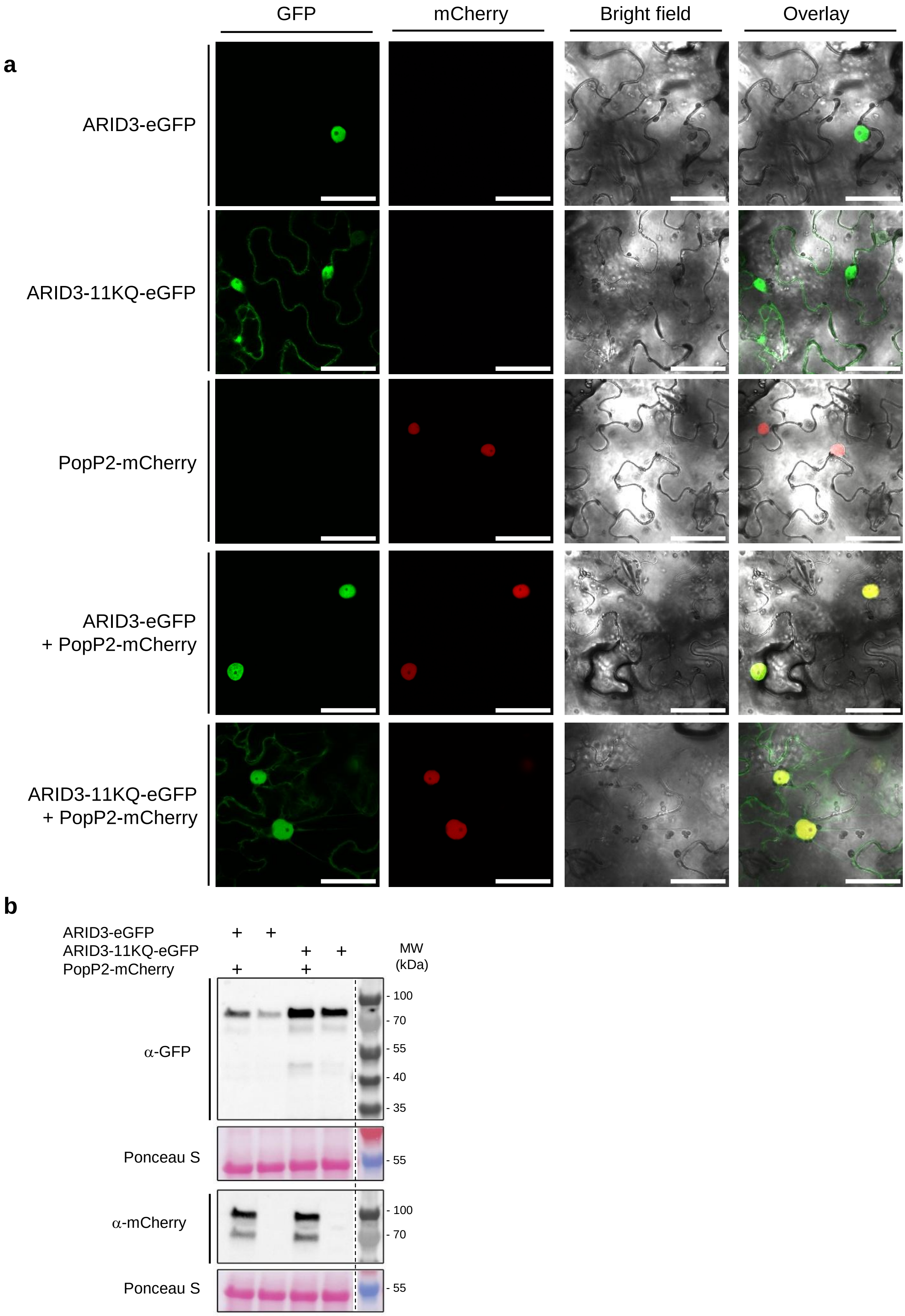

Figure S8. ARID3-11KQ-eGFP is nucleo-cytoplasmic by contrast to ARID3-eGFP that specifically accumulates in the plant nucleus. (a) ARID3-eGFP and ARID3-11KQ-eGFP were transiently expressed in *N. benthamiana* leaves either alone, or with PopP2-mCherry. Confocal fluorescence imaging was performed 48h post agro-infiltration. Scale bars=50µm. (b) The expression of the different fusion proteins in *N. benthamiana* was checked by immunoblot analysis. Samples were harvested at 48h post agro-infiltration. Protein extracts were immunoblotted with anti-mCherry (α-mCherry) and anti-GFP (α-GFP) antibodies. Ponceau S staining of total proteins indicates similar amounts of protein loaded between the different lanes. The dashed line indicates that the original image was cut to remove unnecessary lanes. These experiments were performed at least two times with similar results.
